# Chronic senescent human mesenchymal stem cells as possible contributor to the wound healing disorder after exposure to the alkylating agent sulfur mustard

**DOI:** 10.1007/s00204-020-02946-5

**Published:** 2021-01-25

**Authors:** Simone Rothmiller, Niklas Jäger, Nicole Meier, Thimo Meyer, Adrian Neu, Dirk Steinritz, Horst Thiermann, Michael Scherer, Christoph Rummel, Aswin Mangerich, Alexander Bürkle, Annette Schmidt

**Affiliations:** 1 Bundeswehr Institute of Pharmacology and Toxicology, Neuherbergstraße 11, 80937 Munich, Germany; 2 Molecular Toxicology Group, Department of Biology, University of Konstanz, 78457 Konstanz, Germany; 3 Walther-Straub-Institute of Pharmacology and Toxicology, University of Munich, Goethestr. 33, 80336 Munich, Germany; 4 Department of Traumatology and Orthopedics, HELIOS Amper Clinics, Krankenhausstraße 15, 85221 Dachau, Germany; 5 Department of Orthopedics and Sports Medicine, Wolfart Clinic, Waldstraße 7, 82166 Gräfelfing, Germany; 6 Faculty of Human Sciences, Institute for Sports Sciences, Universität Der Bundeswehr München, Werner-Heisenberg-Weg 39, 85577 Neubiberg, Germany

**Keywords:** Senescence, Mesenchymal stem cells, Wound healing disorder, Chemical warfare agents, Sulfur mustard

## Abstract

Wound healing is a complex process, and disturbance of even a single mechanism can result in chronic ulcers developing after exposure to the alkylating agent sulfur mustard (SM). A possible contributor may be SM-induced chronic senescent mesenchymal stem cells (MSCs), unable to fulfil their regenerative role, by persisting over long time periods and creating a proinflammatory microenvironment. Here we show that senescence induction in human bone marrow derived MSCs was time- and concentration-dependent, and chronic senescence could be verified 3 weeks after exposure to between 10 and 40 µM SM. Morphological changes, reduced clonogenic and migration potential, longer scratch closure times, differences in senescence, motility and DNA damage response associated genes as well as increased levels of proinflammatory cytokines were revealed. Selective removal of these cells by senolytic drugs, in which ABT-263 showed initial potential in vitro, opens the possibility for an innovative treatment strategy for chronic wounds, but also tumors and age-related diseases.

**Supplementary Information:**

The online version contains supplementary material available at 10.1007/s00204-020-02946-5.

## Introduction

Damages in the natural barrier of the skin against the environment set the complex wound healing process into motion, during which the skin and underlying tissue repair themselves (Bukowiecki et al. 2017). Mesenchymal stem cells (MSCs) are multipotent adult stem cells and play an essential role in wound healing by accelerating wound closure, enhancing re-epithelialization, increasing angiogenesis, promoting granulation tissue formation, modulating inflammation, and regulating extracellular matrix remodeling (Lee et al. 2016). MSCs home to damaged tissue, and upon arrival, they exert their therapeutic effects mainly by paracrine signaling via various cytokines, immunosuppressive, growth and differentiation factors (Ranganath et al. 2012; Ma et al. 2014). Because of their self-renewal potential, simple isolation process and expansion in vitro, MSCs are a promising tool in regenerative medicine (Bruder et al. 1998) and multiple studies already showed that MSCs enhance wound healing by accelerating wound closure (Walter et al. 2010; Nie et al. 2011; Rodriguez-Menocal et al. 2015).

Failure in wound healing or some diseases, e.g., diabetes mellitus, lead to chronic wounds, that not only reduce the patients’ quality of life but also create a significant financial burden on the healthcare system (Augustin and Maier 2003). Chronic wounds have also been described after exposure to the alkylating chemical warfare agent sulfur mustard (SM) (Schmidt et al. 2013). Although SM has been banned by the Chemical Weapons Convention, it was most recently used in Syria (Kilic et al. 2018; Sezigen et al. 2019; John et al. 2019). Mainly the easy synthesis and stockpiles still existing in various countries enable acute threats of terroristic attacks, which highlights the importance of developing countermeasures. Despite intensive research, the molecular toxicity of SM is incompletely understood, there is no antidote or prophylaxis available and the treatment is only symptomatic (Etemad et al. 2019). Skin is one of the main target organs of SM and skin lesions occurred in more than 90% of exposed persons (Emadi et al. 2008). In detail, after a characteristic latency period without any clinical presentations, SM causes edema, inflammation, skin blisters and ulceration. Skin lesions may take months to heal and often result in chronic wounds, which may require skin grafting (Schmidt et al. 2018b). Additionally to problematic skin damages after primary healed SM affections, it was discovered during World War I that sites of the body with previous SM exposure which had already partially or entirely healed, became active again in some patients when the same individual was exposed to SM even years later at other, distant skin sites (“flare-ups”) (Sulzberger et al. 1947).

Because MSCs show important properties in wound healing, they may be affected during SM poisoning. This is underlined by the fact that MSCs derived from patients suffering from chronic wound healing disorders showed reduced migratory attraction to skin and wound fibroblasts compared to healthy donors (Rodriguez-Menocal et al. 2012). SM might be able to affect MSCs in bone marrow, as it was shown that SM victims demonstrated bone marrow depletion similar to cytostatic treatment (Hassan and Ebtekar 2002). Indeed, bone marrow identified as was one of the most sensitive tissues in terms of DNA crosslinks (Yue et al. 2015). We could already demonstrate that although MSCs are highly resistant against SM in terms of cell survival, even low doses do reduce the migration (Schmidt et al. 2013) and proliferation but not apoptosis (Schmidt et al. 2018a). We could also show dramatic changes in the secretome and increased migration by addition of specific cytokines (Schreier et al. 2018).

Chronic wounds and flare-ups are long-term complications after SM exposure and thus there needs to be some kind of “memory”, which may be senescent cells due to their longevity. Senescence is part of many cellular mechanisms including aging (Baker et al. 2016), age-related diseases (Baker et al. 2011), tissue remodeling (Krizhanovsky et al. 2008), wound healing (Jun and Lau 2010) and immunity (Kearney et al. 2015). Recently, senescence was defined as a specific permanent growth arrest of proliferation-competent cell induced by stressors (Sharpless and Sherr 2015) like reactive oxygen species (ROS) (von Zglinicki 2002) or unresolved DNA damage (Sedelnikova et al. 2004). Most routinely the expression of senescence-associated β-galactosidase (SA-β-gal), p16^INK4A^ and the senescence-associated secretory phenotype (SASP) are used as biomarkers. Affected cells are not eliminated by the immune system, persist over months or years and secrete proinflammatory factors (Sharpless and Sherr 2015). Thus, senescent MSCs may play a role in the chronic wound healing disorder after SM exposure. A treatment using senolytic drugs, i.e., natural products or small synthetic molecules that selectively eliminate senescent cells, as it has already been successfully tested in human MSCs after replicative senescence (Grezella et al. 2018), could clear senescent MSCs after SM exposure. In this study, we investigated whether SM could induce senescence in MSCs and thereby may influence wound healing as well as whether senolytic drugs are a promising option for treatment of SM induced wounds.

## Results

### Senescence and apoptosis induction in mesenchymal stem cells by sulfur mustard and hydrogen peroxide

**Fig. 1:**
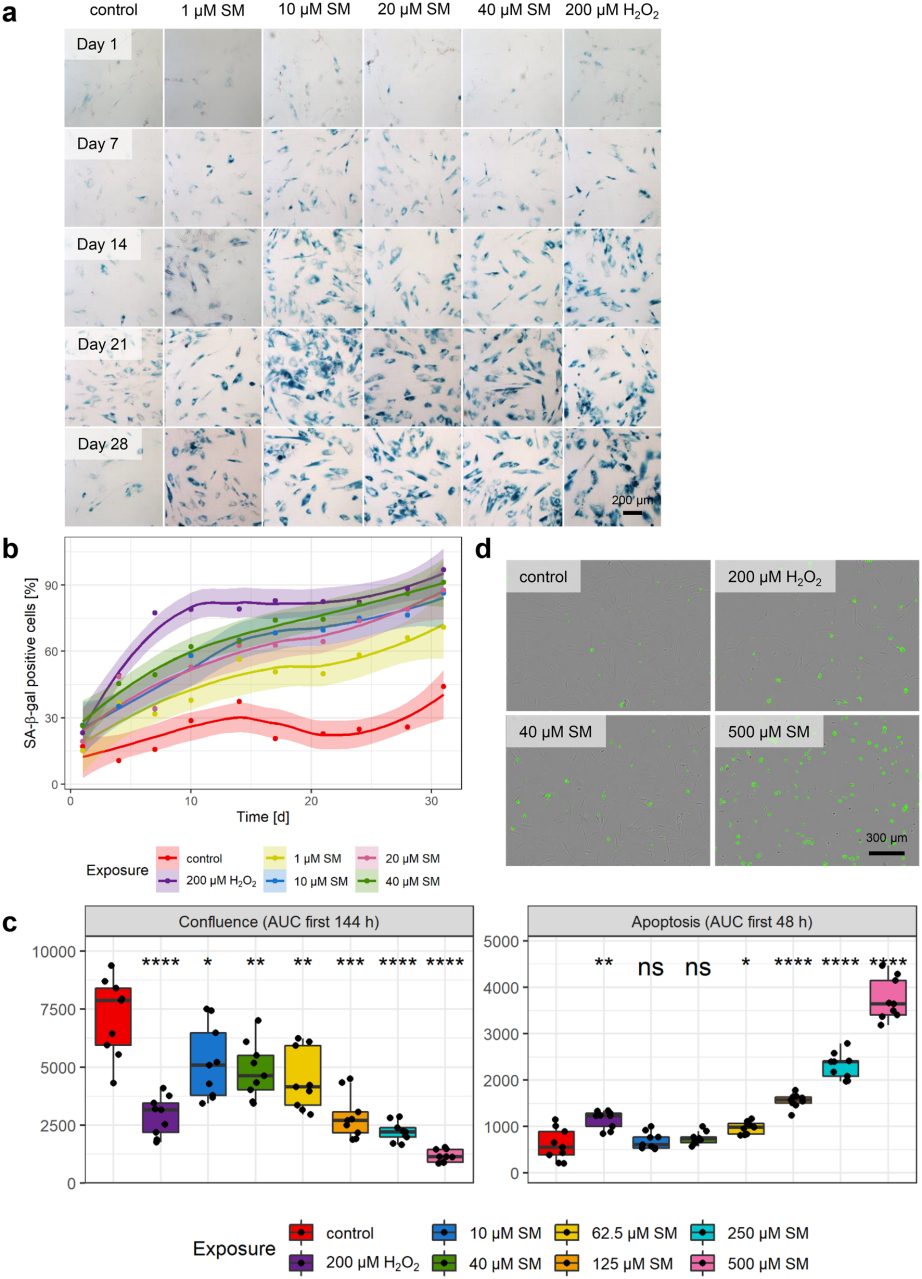
Senescence and apoptosis induction. **a** Representative images of concentration- and time-dependent development of SA-β-gal staining (blue) after single dose SM or H_2_O_2_ exposure (day 0) in contrast to solvent controls. Counterstaining of all cells with nuclear fast red (red). Scale bar, 200 µm. **b** The means with a trend including 99% confidence intervals of the percentage of SA-β-gal positive cells counted every 3–4 days over 31 days (*n* = three randomly selected fields per group from three independent experiments). **c** Area under the curve (AUC) for confluence (area covered by cells) within the first 144 h and apoptosis (**d** annexin V positive cells (green), representative images *t* = 48 h) within the first 48 h. Shown is the significance between solvent controls and 200 µM H_2_O_2_ or SM exposed MSCs (n = three randomly selected images per group from three independent experiments). Data are represented as Tukey boxplots; *p < 0.05, **p < 0.01, ***p < 0.001, ****p < 0.0001. See also Supplementary Figs. 1–3

For all experiments, human bone marrow derived MSCs were used (Supplementary Fig. 1a–h), and their identity was checked regularly by morphology, expression of cell surface markers and differentiation potential (Supplementary Fig. 1i, j). SM concentrations from LC_1_ (1 µM) to LC_25_ (40 µM) (Schmidt et al. 2013) were used initially to test whether a single dose exposure can induce senescence in MSCs. SA-β-gal staining was used to determine the percentage of senescent cells at different time points after exposure and H_2_O_2_ was used as a positive senescence induction control (Brandl et al. 2011). As shown in Supplementary Fig. 1k, cell survival after H_2_O_2_ exposure was determined at the half-maximal lethal concentration (LC_50_) of 322.7 ± 11.4 µM. Therefore, 200 µM H_2_O_2_ (LC_16_) was used for subsequent experiments. To assess the concentration and time dependence of senescence induction, staining was performed up to 31 day post-exposure (day 0), with intervals of 3–4 days. As is shown in Fig. 1a, b, the percentage of senescent cells as well as the staining intensity increased with time. In the concentration range from 10 to 40 µM SM, almost all cells were senescent within 21 days and no obvious change in senescent state was seen thereafter. The exposure to 200 µM H_2_O_2_ showed comparable results, but with a stronger initial increase. In comparison, 1 µM SM did not lead to complete senescence. Increased percentages of SA-β-gal positive cells reached the significance level at days 7, 14, 21 and 28 post-exposure, when compared to solvent controls (Supplementary Fig. 2a). Solvent controls also showed a slight increase in senescence over time, but the conditions were adjusted to keep senescence as low as possible, for example using low passage numbers. For subsequent experiments, cells 21 days after exposure to 10 and 40 µM SM or 200 µM H_2_O_2_, respectively, were considered suitable to be used as senescent cell types and cells exclusively exposed to solvent as non-senescent controls, respectively.

Full replicative senescence occurred not before 13 passages in controls (Supplementary Fig. 3a). Exposure to 40 µM SM resulted in a chronic senescence, since no replication of those cells occurred up to 24 weeks after induction. In comparison, upon 10 µM SM or 200 µM H_2_O_2_ exposure of MSCs, some cells seemed to reenter the cell cycle, based on an increase in cell numbers up to 24 weeks afterwards, but all three exposures resulted still in many SA-β-gal positive cells (Supplementary Fig. 3b). As a proof of principle, we also used commercially available adipose tissue derived MSCs and exposed them to 10, 20 and 40 µM SM or 200 µM H_2_O_2_. After 21 days, many SA-β-gal positive cells could be observed in all groups compared to the solvent control (Supplementary Fig. 3c). Moreover, senescence could also be induced by continuous SM or H_2_O_2_ exposure. While 1 µM SM or 50 µM H_2_O_2_ two to three times per week was sufficient, 0.1 or 0.5 µM SM was too low to result in close to 100% SA-β-gal positive cells after 21 days (Supplementary Fig. 3d). The SA-β-gal positive senescent phenotype of MSCs 21 days after exposure to 40 µM SM was not only comparable to 200 µM H_2_O_2_, but also to exposure with DNA-damaging chemotherapeutic agents like 20 µM cisplatin, 50 µM melphalan and 50 µM bendamustine or ionizing radiation with 10 Gy (Supplementary Fig. 3e).

Since the concentrations used for senescence induction also reduced cell viability, the influence on cell growth and apoptosis was determined. After the exposure to increasing SM concentrations, 200 µM H_2_O_2_ or solvent, the confluence, i.e., area covered by cells, and apoptotic cells, i.e., number of cells positive for Annexin V, were recorded microscopically for more than 11 days. Supplementary Fig. 2b shows that the linear range of confluence increase in the solvent control was up to about 144 h and a maximum of apoptotic cells was reached within about 48 h determined by 500 µM SM exposure. Following these observations, the area under the curve (AUC) was calculated within these time frames for statistical analysis. As shown in Fig. 1c, the confluence was significantly decreased when cells were exposed to 200 µM H_2_O_2_ or SM in a concentration-dependent fashion from 10 to 500 µM, when compared to solvent controls. On the other hand, 200 µM H_2_O_2_ significantly increased apoptosis within 48 h post-exposure in comparison to controls. For SM exposure, 10 and 40 µM did not result in a significant increase, while apoptosis was significantly higher for concentrations between 62.5 and 500 µM SM (Fig. 1c, d). Both confluence reduction and apoptosis increase were very similar from 10 to 62.5 µM SM, and beyond 62.5 µM they were concentration dependent. Interestingly, the SM concentrations 10 and 40 µM resulted in senescence but not apoptosis.

### Senescence markers

**Fig. 2:**
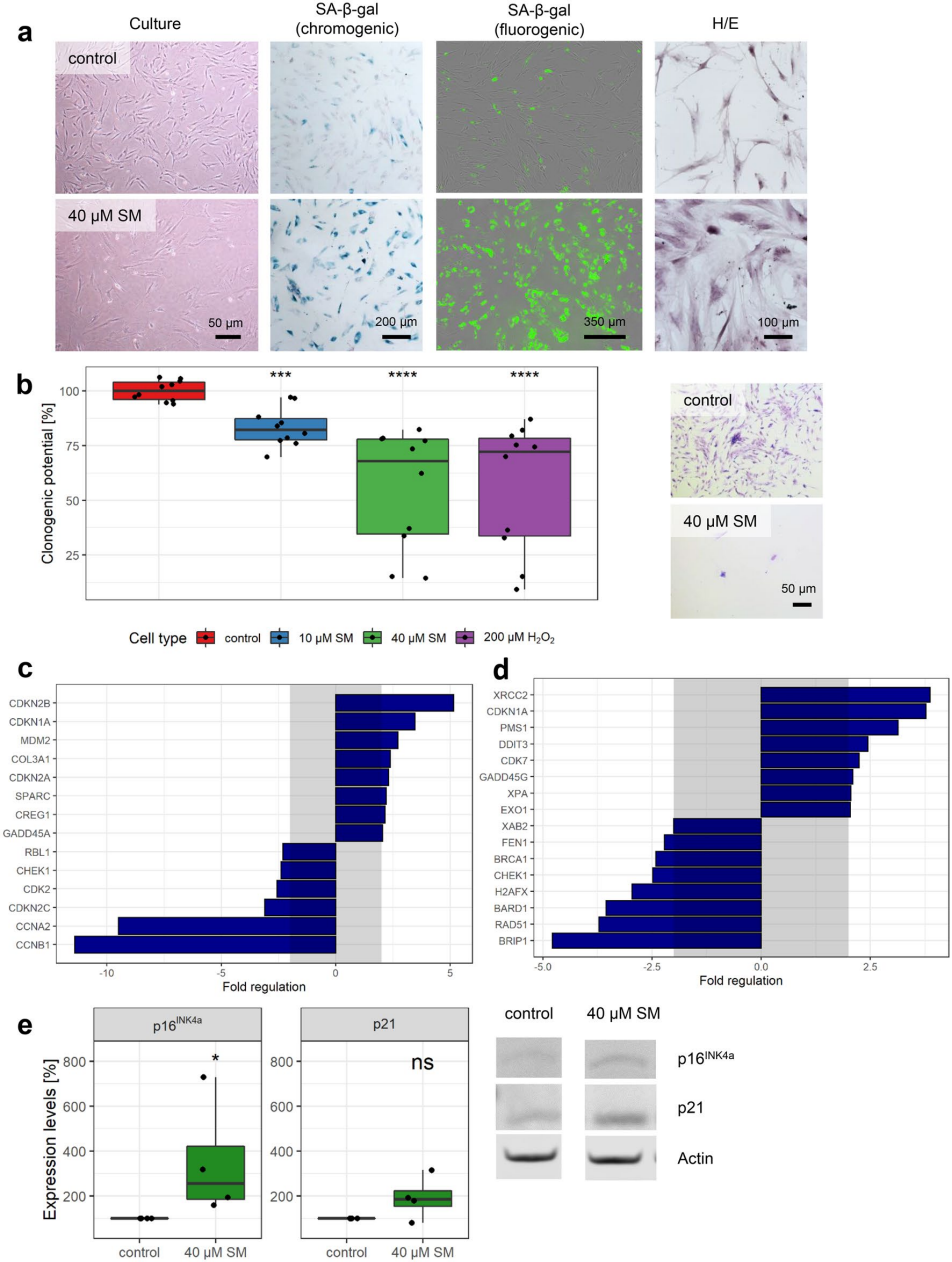
Senescence markers. **a** Representative images from both chromogenic (PromoCell) and fluorogenic (Cell Biolabs) SA-β-gal staining in MSCs 21 days after 40 µM SM exposure show a very high percentage of senescent cells with high staining intensity compared to solvent controls. Flattening and enlargement shown already in culture as well as by hematoxylin/eosin (H/E) staining. **b** Loss of replicative potential shown by clonogenicity assay. Colonies formed and stained with crystal violet could be observed for controls, but only single cells for 40 µM SM senescent cells. After destaining with methanol, crystal violet absorbance examined at 570 nm by plate reader and mean of controls set to 100% (*n* = duplicates per group from five independent experiments). Genes associated with **c** senescence biomarkers or **d** DNA damage and DNA repair were tested 21 days after 40 µM SM exposure and compared to controls. After RNA extraction from MSCs, a RT-qPCR assay was performed and fold regulation of up- or downregulated genes (≥ 2.0 or ≤ − 2.0 in combination with *p* < 0.05, outside grey box) are shown as means (*n* = three independent experiments). **e** The upregulation of p16^INK4a^ and p21 was also observed using Western Blot. Representative bands and expression levels are shown (n = four independent experiments). Data are represented as Tukey boxplots; *p < 0.05, **p < 0.01, ***p < 0.001, ****p < 0.0001. See also Supplementary Fig. 4

To verify that SM-exposed MSCs are senescent, a variety of methods and senescence biomarkers were used. In Fig. 2a, MSCs 21 days after exposure to 40 µM SM showed increased SA-β-gal activity compared to solvent controls by the chromogenic substance X-Gal as well as by a fluorogenic substrate. Morphological changes like cell size increase and flattening could already be observed during culture, but also by H/E staining. The fluorogenic substrate was also used to identify SA-β-gal positive cells by flow cytometry and Supplementary Fig. 4a shows that exposure to 10 and 40 µM SM as well as 200 µM H_2_O_2_ increased the SA-β-gal positive cell population from 4.0% in solvent control to 46.7–58.8% after 3 weeks. Photomicrographs (Supplementary Fig. 4b) showed the increased cell size and granularity of the senescent cells in accordance with higher cell areas and side scatter intensity numbers (Supplementary Fig. 4c). Loss of proliferation of senescent cells could be shown as they had a significantly reduced colony forming (“clonogenic”) potential. Non-senescent controls showed many grown colonies with more than 60 cells, while senescent cells were still mostly single cells only (Fig. 2b).

The expression levels of various senescence marker genes were observed to be significantly different (fold regulation > 2 or < -2 in combination with *p* < 0.05) in senescent cells (40 µM SM 21 days post-exposure) compared to non-senescent controls (Fig. 2c, Supplementary Table 1). Many genes involved in cell cycle control like ‘cyclin-dependent kinase inhibitor 2B (CDKN2B)’, 1A (CDKN1A, p21) and 2A (CDKN2A, p16^INK4a^) were more than 5-, 3- or twofold upregulated, respectively. The ‘mouse double minute 2 homolog (MDM2)’; ‘cellular repressor of E1A-stimulated genes 1 (CREG1)’; ‘collagen, type III, alpha 1 (COL3A1)’; ‘secreted protein, acidic, cysteine-rich (SPARC)’ and ‘growth arrest and DNA-damage-inducible, α (GADD45A)’ were also more than twofold upregulated. Down-regulated genes also included a variety of cell cycle regulators including ‘retinoblastoma-like protein 1 (RBL1)’; ‘checkpoint kinase 1 (CHEK1)’; ‘cyclin-dependent kinase 2 (CDK2)’; ‘cyclin-dependent kinase inhibitor 2C (CDKN2C)’; ‘cyclin A2 (CCNA2)’ and ‘cyclin B1 (CCNB1)’ between 2- and 11-fold downregulation.

Furthermore, the influence of SM-induced senescence on gene expression of DNA damage or repair related genes for persistently upregulated DNA damage response (DDR), another biomarker, was investigated (Fig. 2d, Supplementary Table 1). In senescent cells, besides CDKN1A between 2- and almost fourfold upregulated expression was recorded for ‘DNA-damage-inducible transcript 3 (DDIT3)’; ‘growth arrest and DNA-damage-inducible, γ (GADD45G)’; ‘X-ray repair complementing defective repair in Chinese hamster cells 2 (XRCC2)’; ‘exonuclease 1 (EXO1)’; ‘postmeiotic segregation increased 1 (PMS1)’; ‘*Xeroderma pigmentosum*, complementation group A (XPA)’ and ‘cyclin-dependent kinase 7 (CDK7)’. Interestingly, downregulated genes (by between 2- and almost fivefold) were almost all involved in the repair of DNA double strand breaks. Amongst these, ‘breast cancer 1, early onset (BRCA1)’; ‘BRCA1 associated RING domain 1 (BARD1)’; ‘BRCA1 interacting protein C-terminal helicase 1 (BRIP1)’; ‘H2A histone family, member X (H2AFX)’ and RAD51. Down-regulated genes were also found for other DNA repair mechanisms, besides CHEK1 the ‘XPA binding protein 2 (XAB2)’ and ‘flap structure-specific endonuclease 1 (FEN1)’. No differences in gene expression were observed for poly (ADP-ribose) polymerases 1–3 (PARPs 1–3).

The upregulation of p16^INK4a^ at the gene level corresponds with a significant increase in protein expression determined by western blot. The increase of p21 was significant on the gene, but not on the protein level (Fig. 2e).

### Increase in secreted proinflammatory factors due to senescence

One very important aspect and biomarker of senescent cells is their secretome called SASP (Coppé et al. 2008). Therefore, cells were exposed to 10 or 40 µM SM, 200 µM H_2_O_2_ or solvent control and at different time points thereafter the levels of secreted chemokines, cytokines and growth factors were determined by Bio-Plex assays. At each time point, 72 different factors were tested (Supplementary Fig. 5), and the fold regulation was calculated relative to the corresponding solvent controls. Since many factors were either secreted at low levels in general or the fold regulation was small, specific criteria were applied (> 20 pg/mL in combination with fold regulation > 2 or < − 2).

**Fig. 3:**
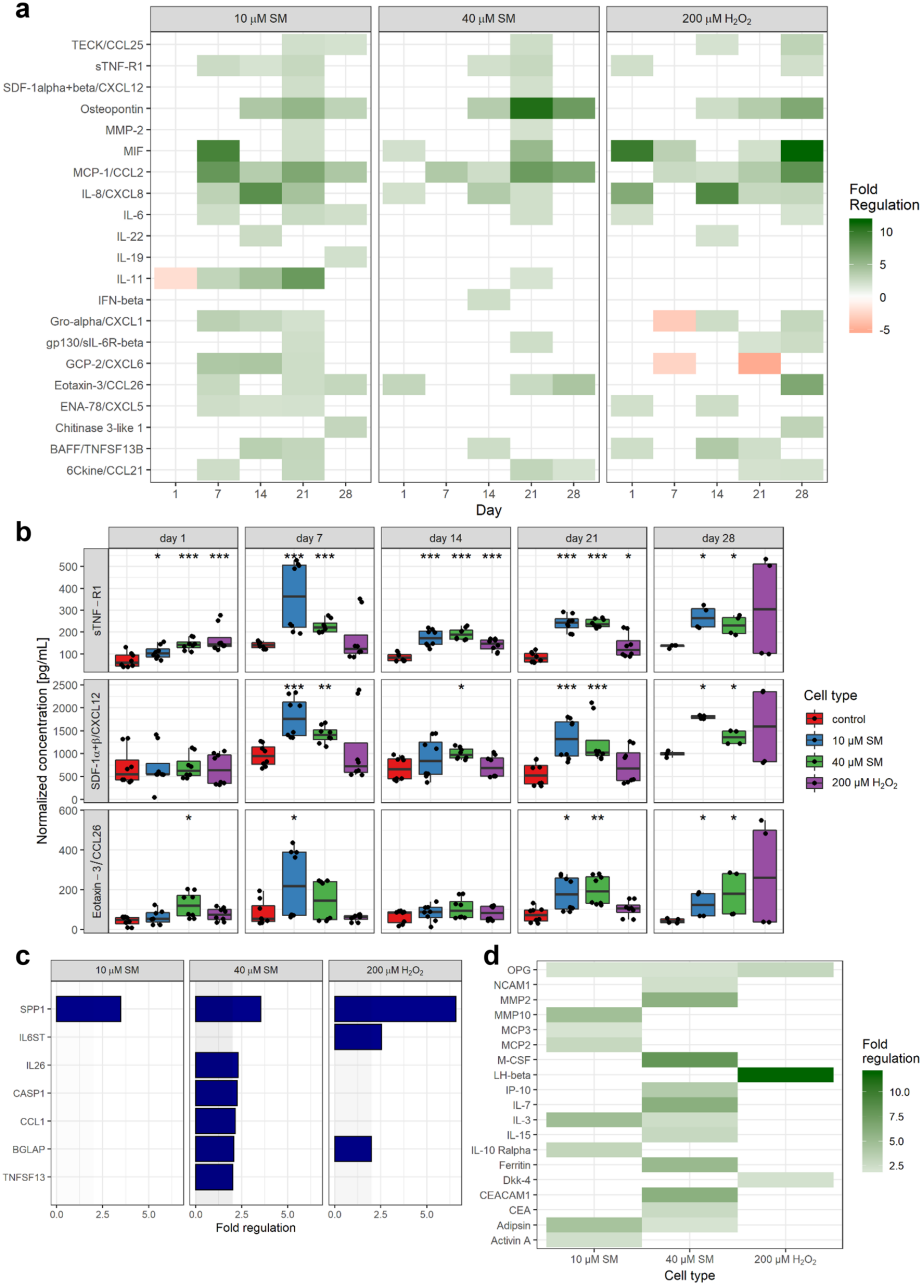
Senescence-associated increase in proinflammatory factors. **a**, **b** The concentration of over 70 chemokines, cytokines and growth factors was determined by Bio-Plex assays in cell culture supernatants and normalized for the cell number (*n* = biological duplicates per group from three independent experiments). **a** Fold regulation of up- or downregulated factors (≥ 2.0 or ≤ − 2.0 in combination with normalized concentrations above 20 pg/mL) of senescent to non-senescent controls are shown. **b** Three factors showed an upregulation in SM- but not in H_2_O_2_-exposed MSCs. **c** After RNA extraction from MSCs at day 21 after exposure to 10 and 40 µM SM, 200 µM H_2_O_2_ or solvent, a RT-qPCR assay of a custom designed cytokine and chemokine gene panel was performed and fold regulation of up- or downregulated genes (≥ 2.0 or ≤ -2.0 in combination with *p* < 0.05, outside grey box) are shown as means (*n* = three independent experiments). **d** Cell culture supernatants were collected 21 day post-exposure and freshly transferred to glass slide based ELISA cytokine arrays. Images were recorded with Odyssey scanner and spot intensities of 279 different factors calculated. After normalization with the positive controls, fold regulation was calculated for each senescent cell type against control and fold regulation of up- or downregulated factors are shown (≥ 2.0 or ≤ -2.0 in combination with normalized concentrations above 5%; *n* = biological duplicates per group from two independent experiments). Data are represented as Tukey boxplots; *p < 0.05, **p < 0.01, ***p < 0.001. See also Supplementary Fig. 5

Figure 3a shows that many factors were upregulated starting from day 14 after exposure. Therefore, the factors TECK, sTNF-R1, osteopontin (OPN), gp130, eotaxin-3 and 6Ckine were considered as late factors and some showed an upregulation over various timepoints and could, therefore, be considered as constant factors, e.g., MIF, MCP-1, IL-8, IL-6, Gro-α, ENA-78 and BAFF. From the above-mentioned factors, some were upregulated already within 24 h or the first week after exposure, such as IL-8. Those could also additionally be considered as early factors. Moreover, the factors sTNF-R1, CXCL12 and eotaxin-3 seem to be upregulated in SM- but not in H_2_O_2_-induced senescent cells (Fig. 3b).

Some of the results were also confirmed on the mRNA level determined by qPCR (Fig. 3c, Supplementary Table 1). Therefore, mRNA was isolated from MSCs 21 days after exposure to 10 and 40 µM SM, 200 µM H_2_O_2_ or solvent. OPN (SPP1) was significantly upregulated more than threefold in SM and sixfold in H_2_O_2_, which is completely in line with the results described above. Similarly, also a twofold upregulation was observed for osteocalcin (BGLAP) in 40 µM SM and H_2_O_2_ exposed cells, gp130 (IL6ST) only in H_2_O_2_ and IL26, CCL1 and APRIL (TNFSF13) only in 40 µM SM exposed cells. In addition to the secretome data, caspase 1 (CASP1) was determined to be twofold upregulated in 40 µM SM.

Using another approach, the levels of over 270 cytokines, chemokines and growth factors were determined by a glass-slide ELISA based array. Figure 3d shows the more than twofold upregulated factors of senescent cells compared to control levels at 21 day post-exposure. The levels of osteoprotegerin (OPG) were found to be upregulated in all senescent cells. In both SM-induced senescent cell types IL-3 and adipsin were upregulated, while LH-β and Dkk-4 were only upregulated in H_2_O_2_-induced senescence. In comparison, NCAM1, MMP2, M-CSF, IP-10, IL-7, IL-15, ferritin, ‘Carcinoembryonic Antigen-Related Cell Adhesion Molecule 1 (CEACAM1)’ and CEA are only upregulated in 40 µM SM senescent cells, but MMP10, MCP3, MCP2, IL-10 Rα and activin A only in 10 µM SM cells. Out of these, the MMP2- and IP-10 upregulation was similar to the results described above.

### Reduced migration and scratch closure ability of senescent cells and influence on healthy MSCs

**Fig. 4:**
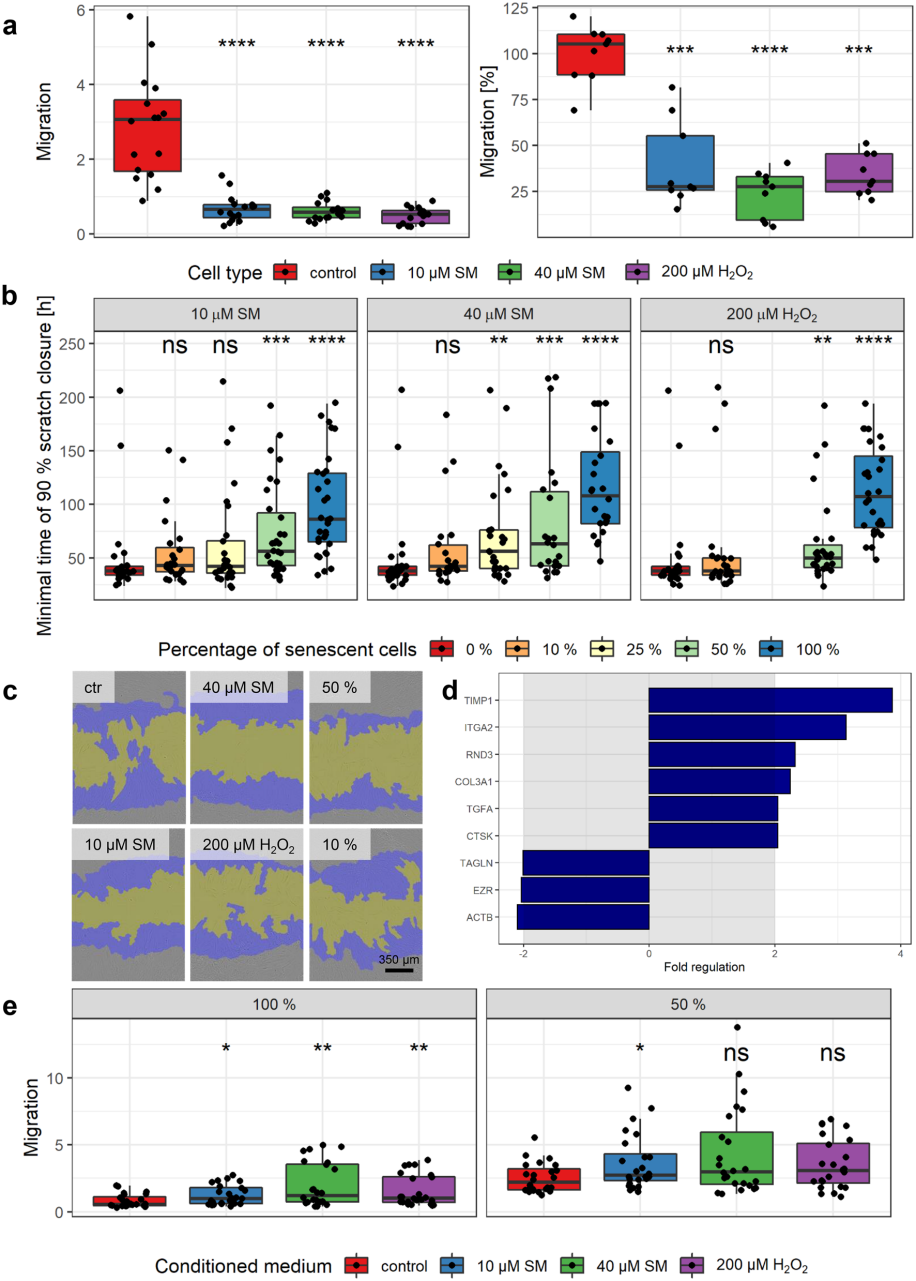
Senescence-associated decreased migration and scratch closure. **a** Senescent cells showed a significant reduction in migration using IncuCyte migration (left) as well as modified Boyden chamber assay (right). Left: Different cells in FBS-reduced medium in inserts migrated towards the reservoir plate filled with standard medium. For normalization, the confluence on the bottom was divided by the confluence on the top for each time point and results are shown after 160 h (*n* = up to 8 biological replicates per group from three independent experiments). Right: Cells were added to the Boyden chamber and incubated for 8 h, fixed, stained with DAPI, and migrated cells were counted. Migration was normalized to the mean of controls (*n *= 3 biological replicates per group from three independent experiments). **b**, **c** Increasing amounts of senescent cells were added to non-senescent controls to observe the influence on scratch closure (0% = only non-senescent solvent controls, 10% = 10% senescent cells + 90% controls, and so on). **b** Scratch assay was performed using the wound maker and IncuCyte microscope, and the minimal time of 90% scratch closure was determined (*n* = up to 8 biological replicates per group from four independent experiments). **c** Representative images at *t* = 24 h (top right 50% and top bottom 10% 40 µM SM, blue initial scratch and yellow cell-free area). **d** Genes associated with wound healing and cell motility were tested in 40 µM SM exposed senescent and non-senescent cells. After RNA extraction from MSCs, a RT-qPCR assay was performed and fold regulation of up- or downregulated genes (≥ 2.0 or ≤ -2.0 in combination with *p* < 0.05, outside grey box) are shown as means (*n* = three independent experiments). **e** The migration of healthy MSCs was increased towards conditioned medium from senescent in comparison to that from non-senescent (control) cells. Conditioned medium was added into the reservoir plate (left) or diluted half and half with culture medium (right). For normalization, the confluence on the bottom was divided by the confluence on the top for each time point and results are shown after 188 h (n = up to 8 biological replicates per group from three independent experiments). Data are represented as Tukey boxplots; *p < 0.05, **p < 0.01, ***p < 0.001, ****p < 0.0001. See also Supplementary Fig. 6

Important properties of MSCs in wound healing are their migration capability. To assess the migration potential, senescent and non-senescent MSCs were allowed to migrate using the chemotaxis assay by IncuCyte. Images were recorded every 2 h (Supplementary Fig. 6a) and for statistical analysis, the migration at the endpoint of the experiment was chosen and shown in Fig. 4a (left). Non-senescent controls showed a high migration potential of about 3, which means three-fold more migrated cells than still present in the upper compartment. All senescent cells showed almost no migration at all, with fewer cells in the lower than the upper compartment (< 1), and was significant, when compared to controls. Using a slightly different technique, i.e., a modified Boyden chamber, migrated cells were counted after 8 h. Hereby also a significantly decreased migration was observed for the senescent cells in comparison to controls (Fig. 4a, right).

The scratch assay was used to study if senescent MSCs are still able to close a scratched area or “wound”. Moreover, different percentages of senescent cells were added to non-senescent controls to see if they would change the time needed to close the scratch. For this assay, the wound maker tool from IncuCyte was used, images were recorded every 2 h and scratch closure was analyzed as the percentage of re-populated scratched area by cells (Supplementary Fig. 6b). For comparison, the minimal time to reach a 90% scratch closure was calculated and results are shown in Fig. 4b, c. Between the non-senescent control (0% senescent cells) with a minimal time of 49.4 ± 40.9 h and the senescent cells (100% senescent cells) with 97.5 ± 46.4 h for 10 µM SM, 116 ± 47 h for 40 µM SM and 112 ± 41 h for 200 µM H_2_O_2_ the scratch closure times were significantly elongated by about twofold. The mixture of 90% controls with 10% senescent cells did not result in a significant increase in the minimal time, but with increasing percentages of senescent cells, the minimal time increased. This increase was significant for the 50:50 mixture of all senescent cells with controls and for 25% 40 µM SM with 75% control.

To understand the underlying mechanisms of this migration and scratch closure reduction, gene expression related to cell motility and wound healing was evaluated by RT-qPCR (Fig. 4d, Supplementary Table 1). Wound healing related genes besides COL3A1 (see 2.2) such as ‘TIMP metallopeptidase inhibitor 1 (TIMP1)’; ‘integrin, α 2 (ITGA2)’; ‘transforming growth factor, α (TGFA)’ and ‘cathepsin K (CTSK)’ were upregulated between 2 - and almost fourfold in 40 µM SM senescent cells compared to controls. The motility related ‘Rho family GTPase 3 (RND3)’ was upregulated twofold. Downregulated by more than twofold were ‘transgelin (TAGLN)’; ‘ezrin (EZR)’ and ‘actin, beta (ACTB)’.

Senescent cells secrete a variety of proinflammatory factors and their conditioned medium increased the migration of tumor cell lines (Minieri et al. 2015). Thus, the influence of conditioned medium onto the migration of healthy low-passage MSCs was studied using the IncuCyte microscope (Supplementary Fig. 6c). For statistical analysis, migration at the endpoint was chosen and shown in Fig. 4e. The migration towards pure conditioned medium (100%, left side) was significantly higher when derived from all senescent cells in comparison to non-senescent controls. To reduce a possible nutrient deficiency in the conditioned medium, it was mixed with the same volume of fresh medium (50%, right side). Using this set-up, only the conditioned medium from 10 µM SM senescent cells resulted in a significant increase compared to medium from controls, but for all a tendency towards an increased migration could be shown.

### Elimination with senolytic drugs

**Fig. 5:**
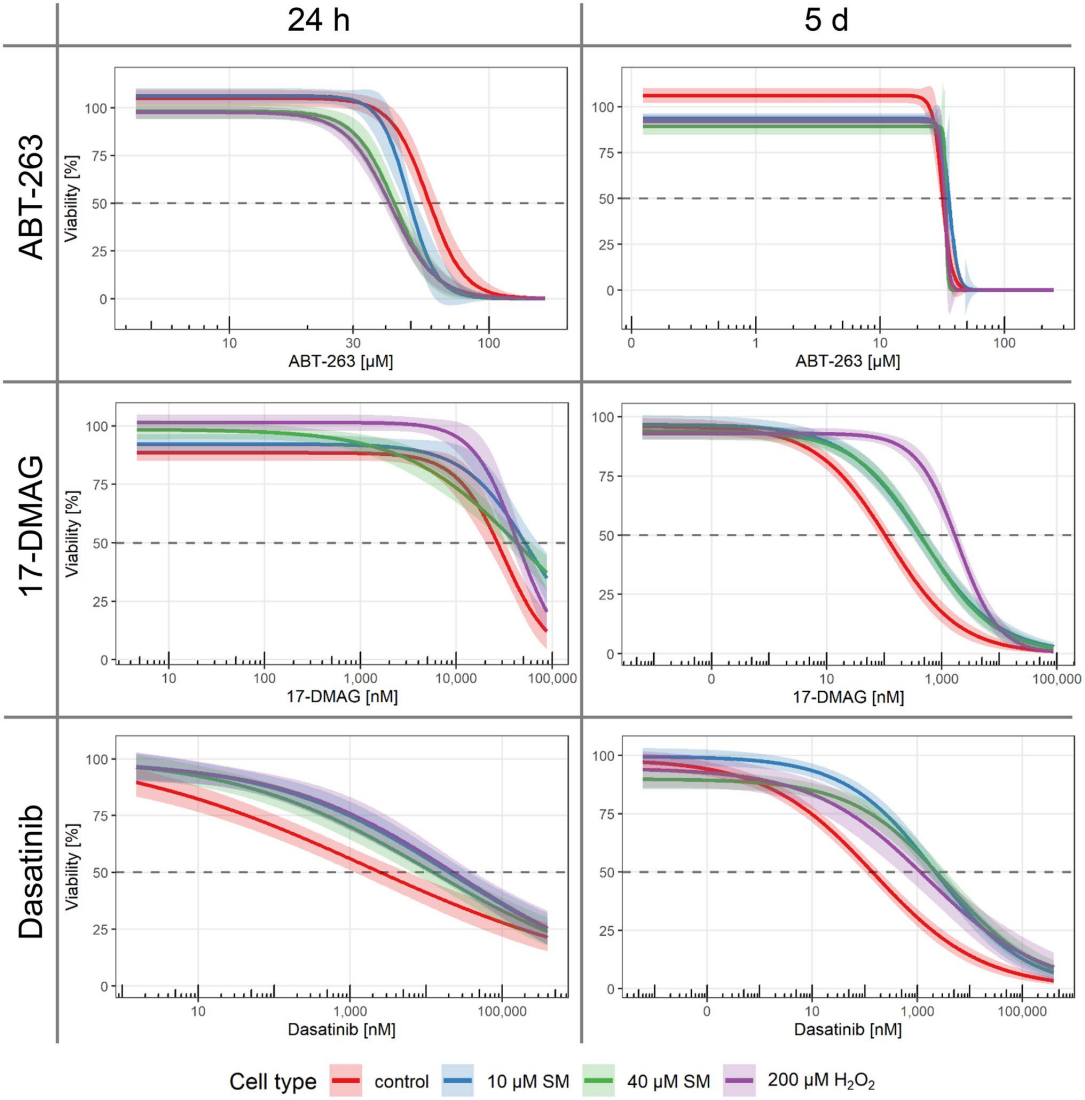
Elimination with senolytics. The selectivity towards senescent cells of literature reported senolytic drugs was tested. Senescent and non-senescent (control) MSC were treated with increasing concentrations of ABT-263, 17-DMAG or dasatinib for 5 days or 24 h. Viability was assessed by XTT assay and normalized for each cell type to the corresponding solvent controls (*n* = 4 biological replicates per group from two or three independent experiments). Data are represented as linear regression including 99% confidence intervals. See also Supplementary Fig. 7

Since senescent cells display various detrimental properties, their targeted and specific elimination might result in improved wound healing, as shown before (Demaria et al. 2014). A total of eight different drugs described as senolytics in the literature were used in increasing concentrations to determine the half-maximal lethal concentration (LC_50_) in senescent (10 and 40 µM SM or 200 µM H_2_O_2_) and non-senescent control MSCs. The senolytics applied were from different groups. Etomoxir and antimycin A target differences between senescent and non-senescent cells in cell metabolism (Dörr et al. 2013); dasatinib and quercetin are kinase inhibitors (Zhu et al. 2015); ABT-737 and ABT-263 are inhibitors of anti-apoptotic BCL proteins (Chang et al. 2016; Yosef et al. 2016); FOXO4-DRI is a synthetic peptide which inhibits the interaction between FOXO4 and p53 (Baar et al. 2017); and 17-DMAG is a HSP90 inhibitor (Fuhrmann-Stroissnigg et al. 2017). First, viability was tested 5 day after the exposure to senolytics and the LC_50_ values including 99% confidence intervals are shown in Table 1. None of the tested drugs showed the desired specificity, while ABT-263, antimycin A, etomoxir and quercetin seemed to affect all cells almost equally with mostly overlapping confidence intervals around the LC_50_. By contrast, the other tested substances, i.e., 17-DMAG, dasatinib, ABT-737 and FOXO4-DRI, displayed a higher specificity for non-senescent cells, based on the lower LC_50_ value than senescent cells. In our hands, ABT-737 was not even able to reduce the viability of senescent cells until the limit of solubility was reached. Since senolytics might not be able to persist and act for as long as 5 days, a shorter time period of 24 h was additionally used for three selected substances and the reduced time span resulted in a tolerance to overall higher concentrations. The substances 17-DMAG and dasatinib showed a comparable result as the non-senescent controls were still more affected. Surprisingly, the reduced time span resulted in the desired effect for ABT-263 with a significantly reduced LC_50_ value for all three senescent cell types compared to non-senescent controls (Table 1). The comparison between the time points is shown in Fig. 5 and other senolytics in Supplmentary Fig. 7.

**Table 1:** LC_50_ values for senolytic drugs. LC_50_ values including the 99% confidence intervals for senolytic drugs after treatment for 5 days and 24 h

Drug	Treatment	Control	10 µM SM	40 µM SM	200 µM H_2_O_2_
17-DMAG [nM]	5 days	118 ± 13	460 ± 66	510 ± 68	1911 ± 148
17-DMAG [nM]	24 h	31,041 ± 2573	59,457 ± 6661	44,276 ± 5,408	42,871 ± 3036
ABT-263 [µM]	5 days	31.6 ± 0.5	36.1 ± 2.2	33.8 ± 1.5	33.1 ± 0.3
ABT-263 [µM]	24 h	58.1 ± 1.6	48.9 ± 0.9	43.4 ± 1.2	41.7 ± 0.9
ABT-737 [µM]	5 days	115 ± 11	*(1054* ± *315)*	*(2,364* ± *1,403)*	*(8536* ± *12,628)*
Antimycin A [µM]	5 days	560.9 ± 92.3	513.3 ± 39.8	613.3 ± 61.5	586.4 ± 99.6
Dasatinib [nM]	5 days	152 ± 25	2259 ± 362	3692 ± 742	1543 ± 604
Dasatinib [nM]	24 h	1690 ± 813	20,773 ± 7103	11,633 ± 4131	24,457 ± 8867
Etomoxir [µM]	5 days	544 ± 21	568 ± 17	584 ± 17	555 ± 23
FOXO4-DRI [µM]	5 days	44.4 ± 1.8	53.1 ± 2.3	57.7 ± 2.1	56.4 ± 2.3
Quercetin [µM]	5 days	116.0 ± 8.7	104.1 ± 5.4	121.1 ± 7.6	177.2 ± 10.6

Italic values in brakes reflect calculations but are above solubility limit

## Discussion

Here we provide insight into a novel pathomechanism after SM exposure, the chronic senescence in human MSCs as possible contribution to the chronic wound healing disorder. Our results show a concentration- and time-dependent senescence induction after single dose SM exposure with deep senescence from 10 to 40 µM SM 21 days later, recorded as intense SA-β-gal staining of all cells. This senescence was chronic for 40 µM SM induced MSCs, since they were still fully senescent even 24 weeks after exposure. As a proof of principle, senescence could also be induced in commercially available adipose tissue derived MSCs. Moreover, senescence could be induced by continuous treatment with lower SM concentrations, and the senescence phenotype by SA-β-gal staining was comparable to MSCs exposed to alkylating cytostatic drugs (Collado et al. 2007) as well as to ionizing radiation (Alessio et al. 2015) in accordance with published studies. For comparison, solvent controls were used as non-senescent MSCs for further studies, since they kept their replicative potential and showed replicative senescence only at passages way higher than those used in experiments, and exposure to 200 µM H_2_O_2_ was used as a positive senescence induction control (Brandl et al. 2011). Our results also suggest that SM exposure of MSCs results primarily in senescence for lower concentrations (up to 40 µM), while higher concentrations mainly lead to apoptosis, as determined by annexin V. This is in line with previous findings of our group revealing that likewise, up to 40 µM SM exposure did not elevate levels of cleaved caspase-3, caspase-8, PARP p85 and apoptosis-inducing factor within 48 h (Schmidt et al. 2018a). In contrast, H_2_O_2_ exposure resulted in both senescence and apoptosis for the applied concentration, already suggesting differences between SM- and H_2_O_2_-induced senescence.

Since senescence was already shown to be induced by unresolved DNA damage (Sedelnikova et al. 2004) or RO S (von Zglinicki 2002), it is not surprising that SM also leads to senescence, as it results in various DNA and RNA mono-alkylation adducts and crosslinks (Zubel et al. 2019) as well as the formation of oxygen free radicals (Brimfield et al. 2012). Moreover, first hints of senescence are described as reduced telomere length in SM exposed Iranian veterans (Behboudi et al. 2018; Behravan et al. 2018) and our group has already discovered acute senescence after SM treatment (Schmidt et al. 2018a). Concluding that the blister-inducing concentration of SM is about 150 µM (Smith et al. 1993) and only 16% reaches the circulation (Cullumbine 1947), this would result in low plasma levels. Even 10 µM SM induced a chronic senescence in MSCs, which highlights a possible transferability of our results to in vivo scenarios.

The chronic senescence induced by SM was substantiated by various biomarkers. SA-β-gal positive cells could be easily determined by flow cytometry using a fluorogenic substrate, a convenient and unbiased technique. Higher area and side scatter values are in line with the observed increase in cell size and granularity already during culture and H/E staining. Our results also show loss of proliferation in senescent cells determined by reduced colony forming potential. Significantly elevated levels of p16^INK4A^ were detected both on the protein and mRNA level, a key regulator of in vitro senescence in human cells. It is absent in unstressed tissues of young animals but highly expressed after certain stresses occurring with tumorigenesis, wounding or aging (Sharpless and Sherr 2015). p21 also showed an increase, which was, however, only significant on mRNA level. The chronic senescence after SM exposure seems to be maintained by a specific upregulation, including also CDKN2B, MDM2, CREG1, COL3A1, SPARC and GADD45A, or downregulation, including CHEK1, CDK2, CDKN2C, CCNA2, CCNB1 and RBL1, of cell cycle regulators. Additionally, the persistently upregulated DDR was found in senescent MSCs before (Minieri et al. 2015), which is in line with our results. Almost all DNA repair mechanisms showed differences, but interestingly, most affected genes seem to be involved in the homologous recombination repair of DNA double strand breaks including XRCC2 and EXO1 upregulation and H2AFX, RAD51, BRCA1, BARD1 and BRIP1 downregulation. This gene expression pattern may include some differences to other senescent cells, which would provide interesting new insight into SM-induced senescence in MSCs.

Another essential biomarker is SASP, a term for the senescence-specific secretome (Coppé et al. 2008). The quality and quantity of SASP factors show differences between cell lines, tissues, species, initial senescence inducing strategies and time after senescence induction (Borodkina et al. 2018). Not only cellular senescence itself but also the SASP is hypothesized to be a dynamic process, divided into (1) acute factors (within 36 h), (2) early phase (initiation of senescence up to 10 days later) and (3) the chronic phase (“mature SASP”, after 2–3 weeks). The secreted factors are thought to create a proinflammatory tissue environment as a paracrine effect and reinforce senescence by autocrine effects, which is influenced by positive feedback loops and various complex regulatory mechanisms (Malaquin et al. 2016; Borodkina et al. 2018). Our results clearly show the upregulation of various proinflammatory factors following senescence development. TECK, sTNF-R1, OPN, gp130, eotaxin-3 and 6Ckine could be considered as late factors. Moreover, MIF, MCP-1, IL-8, IL-6, Gro-α, ENA-78 and BAFF could be considered as constant factors, since they were upregulated during various time points. Out of the factors considered as SASP core for any cell undergoing senescence (Coppé et al. 2008), IL-6, IL-8, Gro-α and MCP-1 but not GM-CSF were also found upregulated in our study. Our results do not confirm GM-CSF as a SASP core factor, which may result from SM as a novel senescence inducer or by human MSCs used as cell type. The absent upregulation of anti-inflammatory cytokines like IL-4, IL-10, IL-13 or IL-35, as typically seen in SASP, is also in line with literature data (Byun et al. 2015). The late factors are of special interest, since they correlate with deep or chronic senescence. For example, OPN is important in immune functions acting as an immune modulator to promote cell recruitment to inflammatory sites, as an adhesion protein involved in cell attachment and wound healing, mediating cell activation and cytokine production, and promoting cell survival as an anti-apoptotic factor (Wang and Denhardt 2008). Moreover, the three factors sTNF-R1, CXCL12 and eotaxin-3 seem to be upregulated in SM-induced, but not in H_2_O_2_-induced senescent cells, which makes them the most interesting factors determined in our study. While sTNF-R1 and eotaxin-3 are not yet described as SASP factors, the literature already displays an importance of CXCL12 in MSCs, senescence and wound healing. CXCL12 is important in angiogenesis and inflammation, and suppression of its activity was suggested to improve scar-free wound healing (Willyard 2018). It is constitutively expressed by MSCs, where it is responsible for the retention of hematopoietic progenitor and stem cells (Janssens et al. 2018) and CXCL12 was largely expressed in senescent tumor cells, where it was related to cancer cell migration and metastasis (Kim et al. 2017). Further research is needed to prove our findings and unravel the underlying mechanisms, eventually resulting in new targets for SM therapy.

Our results show that senescent MSCs have a significantly reduced migratory ability, which was also seen in irradiation induced senescent MSCs (Carlos Sepúlveda et al. 2014). Migration of MSCs is a very important characteristic, since it is essential for homing to damaged tissue to trigger wound healing in vivo (Wang et al. 2014) and this reduction may explain part of its disturbance after SM exposure. Supporting this, the reduced migration resulted in decreased scratch closure ability modeling the wound healing in vitro. Our results showed not only that SM or H_2_O_2_ induced senescent MSCs needed significantly more time to close the scratch, but also the mixture of 50% senescent with 50% non-senescent cells resulted in a significant increase in scratch closure time. This might imply that even a fraction of senescent MSCs might be sufficient to result in the wound healing disorder, since it is unrealistic that all MSCs of an SM exposed victim would become senescent. A panel of cell motility and wound healing genes was analyzed to gain more insight into the underlying mechanisms. In extracellular matrix participating mRNAs of TIMP1, integrin α-2, TGF-α, COL3A1 and cathepsin K were upregulated. The reformation and reorganization of extracellular matrix during wound healing is essential, for example by providing a provisional matrix for the migration of keratinocytes (Thiruvoth et al. 2015). Genes involved in the actin cytoskeleton also seem to play a role in SM induced senescence, since ezrin, its linker to the plasma membrane, the cross-linking transgelin as well as β-actin itself were found to be downregulated, while Rnd3, the regulator in response to extracellular growth factors, was upregulated. Actin filaments as major cell cytoskeleton play important roles in wound healing in vivo, by establishing the initial plug of the wound, where it accumulates at the wound edge, and during remodeling (Abreu-Blanco et al. 2012).

The conditioned medium of senescent cells containing the SASP factors in general and of MSCs in particular was shown to significantly enhance the migration of solid tumor-derived cell lines (Minieri et al. 2015). In our study, we found that the conditioned medium of senescent cells, in contrast to conditioned medium of control cells, enhanced the migration of ‘healthy’ early-passage MSCs. First, this may seem beneficial, since migration is a key property of MSCs (Wang et al. 2014), but on a closer look the healthy MSCs may be more attracted to home to senescent cells than to the site of tissue injury, therefore, resulting in prolonged or disabled wound healing. Moreover, a paracrine transmission of senescence is proposed (van Deursen 2014), already shown for replicative senescent MSCs (Severino et al. 2013), and thus healthy MSCs might also turn senescent by the ‘bystander effect’ (Nelson et al. 2012) resulting in a negative feedback loop.

Taken together, all these detrimental effects of SM-induced MSCs might contribute to the wound healing disorder. Therefore, a treatment using senolytic drugs to selectively eliminate such senescent MSCs would be a novel treatment option. Recent studies raised hopes for regenerative medicine or aging, since the application of senolytic agents was successfully performed in animal studies and intermittent short treatments will have low to no risk of side effects, because senescent cells are unlikely to develop drug resistance as they do not divide (Tchkonia et al. 2013; Zhu et al. 2015). Moreover, senescent cells are increasingly linked to chronic wounds and application of senolytics is discussed as a possible treatment option (Wilkinson and Hardman 2020). In our study, none of the eight senolytic drugs antimycin A, etomoxir, dasatinib, quercetin, ABT-263, ABT-737, FOXO4-DRI and 17-DMAG showed the expected senescence specific effect on cell viability when applied over 5 days. Since ABT-263 exposure of 1 day revealed a senolytic effect in MSCs (Grezella et al. 2018), we additionally tested ABT-263 as well as 17-DMAG and dasatinib with a 24 h exposure. While 17-DMAG and dasatinib were comparable to the longer exposure, ABT-263 exposure of 24 h resulted in the expected lower LC_50_ values of senescent in contrast to non-senescent MSCs. We, therefore, propose ABT-263 as a possible drug candidate.

In conclusion, we demonstrated chronic senescence in human MSCs already after SM single dose exposure. These are impaired regarding proliferation and migration, secrete a variety of proinflammatory cytokines, possibly attract healthy MSCs and may render them senescent. The SM induced senescent MSCs, unable to fulfil their regenerative role, may contribute to the wound healing disorder. An innovative treatment strategy for SM might be the selective clearance of senescent cells by senolytic drugs, an approach in which ABT-263 showed initial potential. Moreover, due to the mechanistic similarity of SM with alkylating cytostatic drugs, our results may also be of interest for therapy-induced senescence (TIS) in cancer patients as well as for the treatment of age-related diseases.

## Materials and methods

### Isolation and subcultivation of MSCs

Human mesenchymal stem cells (hMSCs, henceforth termed MSCs) were isolated from bone marrow of femoral heads, which were donated in the context of total endoprosthesis of the hip joint. They were obtained after obtaining informed consent from patients at the HELIOS Amper Hospital in Dachau, Germany, or Wolfart Hospital in Gräfelfing, Germany. All experiments with human cell material was performed according to authorization of the ethics committee of Ludwig Maximilian University Munich, Germany, and treated as well as disposed according to the requirements of a BioSafety II laboratory. For the experiments, 99 femoral heads were used from donors between 50 and 89 years (mean age 71 years; 46% male and 54% female).

Bone marrow was scraped from the femoral heads using a sharp curette (Allgaier Instrumente GmbH, Frittlingen, Germany), and MSCs were released from trabecular bones by agitation into stem cell medium (HyClone™ minimum essential medium [MEM] alpha modification [GE Healthcare Bio-Sciences Austria GmbH, Pasching, Austria], 20% [v/v] fetal bovine serum [FBS], 400 µM L-glutamine, 100 U/mL penicillin, 100 U/mL streptomycin [all Gibco^®^ by Thermo Fisher Scientific, Waltham, USA]). For separation, the suspension was filtered through a 70 µm cell strainer (BD Falcon™ by Corning, Inc., Corning, USA), and a density gradient centrifugation was performed using Ficoll-Paque™ Plus (GE Healthcare, Chicago, USA) overlaid with the cell suspension for 30 min at 660×*g*, with the brake switched off. MSCs were enriched in the interphase, which was washed once with stem cell medium and centrifuged at 522×*g* for 5 min and plated onto 90 mm cell culture dishes (VWR International, Radnor, USA). Cells were incubated at 37 °C in a humidified atmosphere containing 5% CO_2_ (Thermo Fisher Scientific, Waltham, USA). After 2 days, a medium exchange was performed to eliminate hematopoietic cells. MSCs were further cultured and passaged at about 60% confluence.

Passaging was performed with StemPro^®^ Accutase^®^ Cell Dissociation Reagent (Life Technologies, Carlsbad, USA) for 5 min in the incubator after washing once with PBS. The cell suspension was diluted in medium, pelleted at 522 × g for 5 min, resuspended in fresh medium and counted with Neubauer improved counting chamber (NanoEnTek Inc, Seoul, Korea). Cells were replated at 2000 cells per cm^2^ in fresh medium. Start of experiments was performed up to passage #3. To obtain the best possible comparability, cells were never frozen but instead always used fresh, and plastic labware as well as medium components were kept constant.

### Identification of isolated MSCs

Cells were stained with five different cell surface markers and analyzed via flow cytometry. Briefly, a suspension of 50,000 to 500,000 cells of a low passage number in 1 mL culture medium was stained with the following labeled antibodies for 15 min at room temperature: CD14-FITC (5 µL), CD34-PE-Cy7 (1 µL), CD45-APC-Cy7 (1 µL), CD105-PerCP-Cy5.5 (1 µL) and CD106-APC (5 µL; all Becton Dickinson, Franklin Lakes, USA). An unstained as well as an isotype control using the following labeled antibodies was included: IgG2κ-FITC (5 µL), IgG1κ-PE-Cy7 (5 µL), IgG1κ-APC-Cy7 (5 µL), IgG1κ-PerCP-Cy5.5 (20 µL) and IgG1κ-APC (20 µL; all Becton Dickinson, Franklin Lakes, USA). After staining, cell suspension was washed once, resuspended in annexin binding buffer (Becton Dickinson, Franklin Lakes, USA) and analyzed with BD FACSCANTO Flow Cytometer (Becton Dickinson, Franklin Lakes, USA). MSCs are defined as CD14^−^/CD34^−^/CD45^−^/CD105^+^/CD106^+^.

Moreover, MSCs were also characterized by their potential to differentiate into osteocytes and adipocytes. Therefore, 3.15 × 10^4^ cells per cm^2^ were seeded onto coverslips in 4-well plates. Differentiation medium (PromoCell, Heidelberg, Germany) was changed every 2–3 days for 21 days for osteogenic and 14 days for adipogenic differentiation, respectively. Calcium-rich areas were stained with alizarin red S (Sigma-Aldrich, St. Louis, USA) and lipid drops with Sudan-III (Bio-Optica, Milano, Italy), both with hematoxylin nuclear staining (Bio-Optica, Milano, Italy).

### Viability assessment after hydrogen peroxide exposure

MSCs were plated at 40,000 cells per well in two 24-well plates (Greiner AG, Kremsmünster, Austria) including medium control and grown overnight. The next day, cells were exposed to increasing concentrations of H_2_O_2_ (0.2–80,000 µM) as well as solvent control for 5 days. The 30% (w/w) H_2_O_2_ solution in H_2_O (Sigma-Aldrich, St. Louis, USA) was pre-diluted in ultra-pure water and finally diluted in culture medium. Afterwards cells were washed once with PBS and XTT staining solution (Sigma-Aldrich, St. Louis, USA) was prepared by mixing 5 mL XTT labeling reagent with 100 µL electron-coupling reagent per plate. Cells were incubated with 400 µL medium and 200 µL XTT staining solution, and absorbance was determined at 450 nm with a reference set at 630 nm. Background absorbance was removed using wells only containing medium and viability was normalized to solvent controls. This experiment was performed six times independently (i.e., with cells obtained from six individual donor materials).

### Induction of senescence by sulfur mustard and hydrogen peroxide exposure

Cells were used up to passage three for senescence induction and up to 7 days after last plating (about 70% confluence). Therefore, cells were grown in T175 flasks (Greiner AG, Kremsmünster, Austria).

SM (bis-[2-chloroethyl]sulfide; purity > 99%, confirmed by NMR) was made available by the German Ministry of Defense. For the initial senescence induction study, SM concentrations of 1, 10, 20 and 40 µM, while for later experiments only 10 and 40 µM were used. SM was pre-diluted in pure EtOH and finally diluted in culture medium with a maximum of 0.5% EtOH. Solvent controls were treated with the same volume of pure EtOH. H_2_O_2_ was used as positive senescence control at 200 µM final concentration. The 30% (w/w) H_2_O_2_ solution in H_2_O (Sigma-Aldrich, St. Louis, USA) was once pre-diluted and finally diluted both in culture medium.

For SM and H_2_O_2_ exposed cells, culture medium was changed once a week without passaging of the cells. Solvent controls were sub-cultured once a week by plating 2000 cells per cm^2^. Senescent (10 µM SM, 40 µM SM and 200 µM H_2_O_2_) and non-senescent cells (solvent control) were used for further experiments 21 days after exposure unless otherwise stated.

### Time- and concentration dependence of senescence induction by SA-β-gal staining

After exposure to 1, 10, 20 and 40 µM SM or 200 µM H_2_O_2_, senescence development was checked every third to fourth day using the senescence detection kit I (PromoCell, Heidelberg, Germany) according to manufacturer’s protocol. To exclude false positive staining, cells were plated at the same density 1 day before staining onto cover slips in 4-well plates. Briefly, 10,000 cells were grown overnight on coverslips in 4-well plates, washed once with PBS and fixed for 10–15 min at room temperature with the fixative solution. Meanwhile, the senescence staining solution was prepared by mixing 470 µL of staining solution, 5 µL of staining supplement and 25 µL of 20 mg/mL X-gal in DMSO per well. Fixed cells were washed twice with PBS and senescence staining solution was added and developed in the incubator overnight. Additionally, cells were counterstained with nuclear fast red (Vector Laboratories, Inc., Burlingame, USA) for 10 min at room temperature and mounted with VectaMount™ AQ mounting medium (Vector Laboratories, Inc., Burlingame, USA) onto microscopy slides (Thermo Fisher Scientific, Waltham, USA). Senescence development was monitored over a time period of 4 weeks. The percentage of senescent versus total cells was counted in three randomly selected images (mean of 20 cells per image) and was performed in three independent experiments.

### Replicative senescence

To assess replicative senescence, MSCs were re-plated at 2000 cells per cm^2^ once a week in flasks or culture plates and onto coverslips for SA-β-gal staining (see above). This was performed until the cells did not replicate anymore and no more sub-cultivation was possible, since all cells were used for staining. Experiment was performed three times independently.

### Stability of SM- and H_2_O_2_-induced senescence

The stability of the induced senescence by 10 and 40 µM SM as well as 200 µM H_2_O_2_ was determined by SA-β-gal staining each 5 weeks. Therefore, medium exchange was performed once a week, and every 5 week cells were passaged and 10,000 cells each were used for staining, while the remaining cells were further cultivated. The last staining was performed 24 weeks after exposure and experiment was performed three times independently.

### Time- and concentration dependence of senescence induction in commercially available MSCs

The same experiment as described in 4.5 was performed with commercially available normal human adipose-derived MSCs and the recommended MSC basal medium plus MSC growth kit—Low serum (all from American Type Culture Collection [ATCC], Manassas, USA). Briefly, one vial was thawed, and cells were plated at 2000 cells per cm^2^ and subcultured once a week using the same procedure as for bone marrow derived MSCs. After exposure to solvent control, 10 and 40 µM SM or 200 µM H_2_O_2_, SA-β-gal staining was performed every 3–4 days up to day 31. Experiment was performed two times independently.

### Senescence induction by continuous exposure and other senescence inducers

It was also investigated whether continuous SM and H_2_O_2_ treatment induced senescence. Therefore, MSCs were exposed to 0.1 µM, 0.5 µM and 1 µM SM as well as 50 µM H_2_O_2_ two to three times a week and re-plated for sub-cultivated and SA-β-gal staining once a week up to day 42. Experiment was performed two times independently.

MSCs were exposed to 20 µM cisplatin, 50 µM melphalan or 50 µM bendamustine (all Sigma-Aldrich, St. Louis, USA). Stock solutions of 40 mM cisplatin in DMSO, 33 mM melphalan in acidic EtOH and 40 mM bendamustine in ultrapure water were prepared and then diluted in culture medium. Cells were irradiated at RT with a dose rate of 1 Gy per min with 240 kV x-rays at 13 mA (YXLON Maxishot, Hamburg, Germany) filtered with 3 mm beryllium for 10 min (10 Gy). SA-β-gal staining was performed twice a week up to day 23 and the experiment was performed two times independently.

### Confluence and apoptosis

Cells were plated at 8000 cells per well in a 24-well plate and incubated overnight. The next day, cells were exposed to solvent or SM concentrations of 10 µM, 40 µM, 63 µM, 125 µM, 250 µM and 500 µM or 200 µM H_2_O_2_. After 1 h, annexin V green reagent (IncuCyte by Sartorius, Göttingen, Germany) was added in a final dilution of 1:400. The plate was transferred into the IncuCyte^®^ S3 Live-Cell Analysis System (Sartorius, Göttingen, Germany) and after pre-warming, the first image was recorded about 1.5 h after exposure. Images were recorded every 2 h up to 276 h and the experiment was performed with 3 biological replicates per group and three times independently. Confluence and apoptotic cells were determined by the IncuCyte^®^ S3 Software (Sartorius, Göttingen, Germany).

### Cell morphology by HE staining

20,000 cells were plated onto coverslips in 4-well plates and grown overnight. After washing once with PBS, cells were fixed with 4% PFA in PBS for 30 min at 4 °C. Slips were washed three times with PBS, once with ultra-pure water and stained with 500 µL Mayer’s hematoxylin (Bio-Optica, Milano, Italy) for 5 min at room temperature. To develop the staining, all wells were washed three times with 500 µL tap water followed by once with ultra-pure water. Eosin Y 1% aqueous solution (Bio-Optica, Milano, Italy) was added with 500 µL per well and stained for 3.5 min at room temperature. After washing twice with ultra-pure water, stained cells were dehydrated by dipping the coverslips three times into 90% EtOH and 12 times into xylenes (Sigma-Aldrich, St. Louis, USA) and mounted onto microscopy slides (Thermo Fisher Scientific, Waltham, USA) using Entellan Neu mounting medium (Merck, Darmstadt, Germany).

### Fluorogenic SA-β-gal staining and flow cytometric analysis

For fluorogenic staining, 100,000 senescent or non-senescent cells were plated per well into a 24-well plate and grown overnight. Staining was performed with the quantitative cellular senescence assay kit (Cell Biolabs, Inc., San Diego, USA) according to manufacturer’s protocol. Briefly, culture medium was removed, 2 mL of 1x cell pretreatment solution (1:1000 dilution of 1000x stock in culture medium) was added and incubated for 2 h. After adding 10 µL of 200x SA-β-gal substrate solution directly to the cells, incubation was performed overnight. After washing three times with 3 mL PBS, one image was recorded using the IncuCyte^®^ system and afterwards, cells were detached, resuspended in PBS + 2% FBS and 10,000 cells each analyzed with the ImageStreamX MarkII instrument (Luminex Corporation, Austin, USA). Software used for data acquisition was ISX and for data analysis IDEAS (both Luminex Corporation, Austin, USA). The experiment was performed three times independently.

### Differences in gene expression via qRT-PCR

Non-senescent and 40 µM SM-induced senescent cells each from three individual donor materials were re-plated and 3 days later (i.e*.,* 21 day post-exposure) detached by cell scraper (Greiner AG, Kremsmünster, Austria) directly into the culture medium. For cytokine gene expression analysis, 40 µM SM and 200 µM H_2_O_2_-induced senescent cells were additionally prepared. Cells were pelleted and total RNA was directly extracted according to manufacturer’s protocol using RNeasy Mini Kit (Qiagen, Hilden, Germany). Briefly, the cell pellet was disrupted by adding 350 µL of buffer RLT, vortexed for 1 min, lysate was transferred into a QIAshredder spin column (Qiagen, Hilden, Germany) and centrifuged 2 min at full speed. 350 µL of 70% EtOH was added to the homogenized lysate and mixed by pipetting. The mixture was added to a spin column and centrifuged for 30 s at full speed. The column was washed three times, first with 700 µL buffer RW1 (30 s) and second (30 s) and third time (2 min) with 500 µL buffer RPE each. Spin column was centrifuged dry for 1 min at full speed. RNA was eluted twice with 20 µL nuclease free water (Qiagen, Hilden, Germany) for 1 min at full speed into a biopure 1.5 mL tube (Eppendorf AG, Hamburg, Germany) and stored at − 80 °C.

RNA concentration was determined by NanoQuant with plate reader infinite M200 Pro (Tecan Group AG, Männedorf, Switzerland). For each qRT-PCR plate, 500 ng RNA were separately reverse transcribed into cDNA using RT^2^ First Strand Kit (Qiagen, Hilden, Germany) according to manufacturer’s protocol. Briefly, genomic DNA was eliminated with 2 µL buffer GE, RNA and total volume of 10 µL was obtained with nuclease free water in 0.2 mL PCR-tubes (Eppendorf AG, Hamburg, Germany). Mixture was incubated for 5 min at 42 °C and then immediately incubated on ice for at least 1 min. The reverse transcription mix was prepared by mixing 4 µL 5x Buffer BC3, 1 µL Control P2, 2 µL RE3 Reverse Transcriptase Mix and 3 µL RNase-free water per sample. This mix was added to the 10 µL genomic DNA elimination mix, mixed by pipetting, incubated for 15 min at 42 °C and reaction stopped by incubation for 5 min at 95 °C. All incubations were performed with the Mastercycler nexus GX2 (Eppendorf AG, Hamburg, Germany). After adding 91 µL RNase-free water to each tube, samples were stored at − 20 °C for a maximum of 1 week.

The following RT^2^ Profiler™ PCR Arrays were used: Human Cell Motility, Human Cellular Senescence, Human DNA Damage Signaling Pathway, Human DNA Repair, Human Wound Healing (Qiagen, Hilden, Germany). Additionally, a custom designed array was used to detect various cytokine, chemokine or growth factor gene transcripts (Qiagen, Hilden, Germany). For each plate, 102 µL of one cDNA template was diluted with 1284 µL nuclease free water and placed into the Freedom Evo automated pipetting system with EVOware Standard software (Tecan Group AG, Männedorf, Switzerland) together with one tube of RT^2^ SYBR Green ROX qPCR Mastermix (Qiagen, Hilden, Germany) and the PCR array plate. After extensive treatment with 7% sodium hypochlorite (Carl Roth, Karlsruhe, Germany) and washing with ultra-pure water of the tips, 1350 µL mastermix were added to the diluted cDNA. The mixture was briefly vortexed and for each well, 25 µL of the cDNA-mastermix mixture were added. Before, in between and after the addition, tips were extensively washed with ultra-pure water. The plate was sealed and run on Eppendorf Mastercycler^®^ epgradient S realplex^2^ with Mastercycler ep realplex software (both Eppendorf AG, Hamburg, Germany) with initial activation of polymerase for 10 min at 95 °C followed by 40 cycles of 15 s at 95 °C and 1 min at 60 °C. Before data were exported, threshold was manually set to 200 and the drift correction was activated for better comparison. All experiments were performed three times independently.

C_T_ values were exported and analyzed by GeneGlobe (http://www.qiagen.com/geneglobe) using C_T_ cut-off of 30, RPLP0 housekeeping gene for normalization, fold regulation cut-off of 2 and *p* value cut-off of 0.05. Shown are the fold changes of senescent to control levels obtained.

### Western blot

For western blots, senescent and non-senescent MSCs were plated at 10^5^ cells per well in 6-well plates, and 2 days later cells were extracted. Cells were washed three times with PBS and 100 µL extraction buffer (6.25 mM Tris–HCl pH 7.5, 12.5 mM NaCl, 2.5 mM EDTA, 1.5% Triton X-100, one Complete Mini Inhibitor Cocktail [Roche, Basel, Switzerland] and one PhosSTOP [Roche, Basel, Switzerland] in ultra-pure water) were added per well and cell lysis performed for 15 min on ice. All further steps were performed on ice. Cells were scraped using a cell scraper (Greiner AG, Kremsmünster, Austria), cell suspension transferred into a centrifuge tube (Eppendorf AG, Hamburg, Germany), vortexed and disrupted by ultrasonic homogenizer (Bandelin electronic, Berlin, Germany) three times for 10 s, 0.3 interval and 30% intensity. To complete cell disruption, incubation in extraction buffer was performed for 1 h in total with vortexing several times in between. Cell debris was eliminated by centrifugation at full speed for 10 min at 4 °C. Cell lysates were divided in 25 µL aliquots and stored at − 20 °C.

Cell lysates were denatured by adding 8 µL loading buffer (60 µL 4x protein loading dye [LI-COR Biosciences, Lincoln, USA] and 40 µL 3.12 mg DTT [Sigma-Aldrich, St. Louis, USA] in ultra-pure water) per aliquot and incubation for 5 min at 95 °C. Denatured cell lysates and 3 µL Cameleon™ Duo Pre-stained Protein Ladder (LI-COR Biosciences, Lincoln, USA) were separated on NuPAGE 4–12% Bis–Tris gels 1.0 mm × 10 wells with NuPAGE MES SDS Running Buffer (both Novex by Thermo Fisher Scientific, Waltham, USA) for 60 min at 50 mA using a Mini Gel Tank (Invitrogen by Thermo Fisher Scientific, Waltham, USA). Immobilon^®^-Fl PVDF Membrane with 0.45 µm pore size (Merck, Darmstadt, Germany) was activated by rinsing with methanol (Sigma-Aldrich, St. Louis, USA) and transfer was performed with NuPAGE Transfer Buffer and 500 µL NuPAGE Antioxidant (both Novex by Thermo Fisher Scientific, Waltham, USA) for 90 min at 100 V and 300 mA using Mini Blot Module (Invitrogen by Thermo Fisher Scientific, Waltham, USA).

All subsequent steps were performed with gentle rocking and washing three times with PBS-T (0.1% Tween20 in PBS) for 10 min each. Membranes were blocked for 30 min in Odyssey^®^ PBS Blocking Buffer (LI-COR Biosciences, Lincoln, USA). Primary antibodies were diluted in antibody diluent (Odyssey Blocking buffer with 0.1% Tween20) and membranes were incubated overnight at 4 °C. The following primary antibodies were used: Rabbit Anti-CDKN2A/p16INK4a (ab81278 abcam, Cambridge, UK) 1:2000, Purified Mouse Anti-p21 (556,431 BD Pharmingen by Becton Dickinson, Franklin Lakes, USA) 1:250. After washing, secondary antibody IRDye^®^ 800CW Goat Anti-Mouse or IRDye^®^ 800CW Goat Anti-Rabbit (LI-COR Biosciences, Lincoln, USA) were added 1:8000 in antibody diluent for 90 min at room temperature. After washing, the membrane was developed on Odyssey Clx with Image Studio software (LI-COR Biosciences, Lincoln, USA). For normalization, anti-actin (sc-8432 Santa Cruz, Santa Cruz, USA) primary antibody was used 1:5,000 in antibody diluent and secondary IRDye^®^ 680RD Goat Anti-Mouse (LI-COR Biosciences, Lincoln, USA) as described above. Samples of four independent experiments were used.

### Clonogenic potential

1000 cells of senescent and non-senescent MSCs were seeded per well in a 6-well plate and grown without medium exchange for 10 days. After washing with PBS, fixation with 4% PFA in PBS for 30 min at 4 °C, again washing with PBS and twice with ultra-pure water, colonies were stained with 1 mL of 1% aqueous crystal violet solution (Sigma-Aldrich, St. Louis, USA) for 2 min at room temperature. After extensive washing with ultra-pure water, plates were air dried and pictures of colonies were taken with Nikon Eclipse TS100 microscope (Nikon, Minato, Japan). Afterwards, each well was destained with 500 µL methanol for 20 min at room temperature and 150 µL each were transferred to two wells of a 96-well plate and absorbance at 570 nm was read by plate reader infinite M200 Pro (Tecan Group AG, Männedorf, Switzerland). The absorbance of five independent experiments was normalized to corresponding controls.

### Migration analysis

1000 senescent and non-senescent cells were seeded in their standard medium into a modified Boyden chamber with 24-well FluoroBlok™ cell culture insert system containing 8 µm pores (Corning, Inc., Corning, USA) in 24-well plates (BD Falcon™ by Corning, Inc., Corning, USA). Migration was performed for 8 h and cells were fixed with 4% PFA in PBS for 30 min at 4 °C. The membrane was cut, transferred to a coverslip, and mounted using mounting medium containing 4,6-diamidino-2-phenylindole (DAPI, Vectashield, Vector Laboratories, Inc., Burlingame, USA). The number of migrated cells was counted microscopically (Nikon, Minato, Japan) with three biological replicates per group and the experiment was performed three times independently.

Migration was also assessed using the IncuCyte S3 system (Sartorius, Göttingen, Germany). Briefly, senescent and non-senescent cells were detached, washed once with PBS and resuspended in medium containing only 1% FBS. 1000 cells in 60 µL were seeded per well into the IncuCyte ClearView insert plate (Sartorius, Göttingen, Germany) containing pores of 8 µm diameter, and cells were left to settle at room temperature for 20 min. Then, 200 µL standard culture medium containing 20% FBS was added per well into the reservoir plate (Sartorius, Göttingen, Germany). The insert plate was put on top and imaging was started 30 min after placing into the instrument and then every 2 h for 160 h in total. Using the IncuCyte Chemotaxis analysis software, a mask was designed to recognize cells at the top (non-migrated) and bottom (migrated) of the insert-plate. To exclude proliferation, the covered area of the bottom was divided by the covered area of the top. The experiment was performed with 8 wells per condition and three times independently.

### Wound closure by scratch assay

17,000 cells in 200 µL were seeded in total per well into an ImageLock 96-well plate (Sartorius, Göttingen, Germany), left to settle at room temperature for 20–30 min and incubated overnight. To study different fractions of senescent cells vs. non-senescent cells, 10%, 25% and 50% were added together with 90%, 75% and 50% non-senescent cells, respectively. The scratch was done with the IncuCyte WoundMaker (Sartorius, Göttingen, Germany) according to manufacturer’s protocol. Briefly, the WoundMaker was washed in sterile ultra-pure water and sterilized in 80% EtOH, both for 5 min. Meanwhile, medium was aspirated and 100 µL fresh medium per well added. The plate was inserted into the WoundMaker and a uniform 700–800 µm scratch was created simultaneously in all 96 wells. The medium was aspirated, the scratched cells washed away twice with 100 µL PBS per well and finally 200 µL fresh medium was added. The plate was placed into the IncuCyte S3 (Sartorius, Göttingen, Germany) and first image was recorded 30 min later, then every 2 h up to 234 h. The WoundMaker was cleaned by a series of washing steps with 0.5% Alconox^®^ (Sigma-Aldrich, St. Louis, USA), 1% Virkon S (Antec International Limited, Sudbury, UK), sterile ultra-pure water and 80% EtOH each for 5 min before storage. Using the IncuCyte Scratch Wound analysis software, a mask was designed to recognize the wounded area as well as the cell-covered surface. The percentage of the wound closure was determined with 8 wells per condition from four independent experiments.

### Secretome analysis by Bio-Plex

For the generation of the samples, MSCs were seeded at 2.1 × 10^4^ cells per cm^2^ in a 24-well plate and allowed to attach overnight. The medium was removed and replaced with 600 µL fresh medium supplemented or not with EtOH / SM / H_2_O_2_. Exactly 24 h later, the supernatant was centrifuged at 4 °C and 206 × g for 15 min, divided at 140 µL each in four 0.5 mL Protein LoBind Tubes (Eppendorf AG, Hamburg, Germany) and stored at −80 °C. Samples were taken at days 1, 7, 14, 21 and 28 after exposure.

The samples were analyzed using the Bio-Plex Pro™ Human Chemokine 40-plex Assay and Bio-Plex Pro™ Human Inflammation 37-plex Assay (BioRad, Hercules, USA) according to manufacturer’s protocol. All incubation steps were performed, while plate was covered with provided aluminum foil at room temperature and at 850 rpm on IKA^®^ MS3 digital shaker (IKA, Staufen, Germany). Washing steps were performed three times with 100 µL wash buffer per well using the wash station HydroFlex (Tecan Group AG, Männedorf, Swiss). Briefly, 50 µL beads were added per well into the Bio-Plex^®^ 96-well plate and washed. Standards were diluted as described in cell culture medium and 50 µL were added per well as well as 50 µL of the samples generated. The plate was incubated for 1 h. After washing, 50 µL detection antibodies were added per well and incubated for 30 min. After washing, 50 µL SA-PE were added per well and incubated for 10 min. After the final washing, beads were resuspended in 125 µL assay buffer by shaking for 30 s. Plates were analyzed by the Bio-Plex^®^ 200 System and Realplex software (BioRad, Hercules, USA). The Experiment was performed in biological duplicates per group and three times independently.

### Secretome analysis by cytokine array

For sample generation, 42,500 non-senescent or senescent (10 µM SM, 40 µM SM and 200 µM H_2_O_2_) MSCs were seeded per well in a 24-well plate and grown overnight. Medium was removed and replaced with 1000 µL fresh medium. After exactly 24 h, the supernatant was removed, centrifuged at 24,696 × g at RT for 10 min and the supernatant immediately used as sample.

All samples were analyzed with RayBio^®^ Human Cytokine Antibody Array G-Series 6, 7, 8, 9 and 10 (RayBiotech, Peachtree Corners, USA) according to manufacturer’s instructions. Briefly, array slides were transferred to RT and thawed for 15 min. Arrays were opened, cover removed and dried for 2 h in a laminar flow hood. All incubation steps were performed at room temperature and with 850 rpm on IKA^®^ MS3 digital shaker (IKA, Staufen, Germany), while arrays were sealed with provided adhesive film for steps > 2 h or < 100 µL per well. 100 µL blocking solution (0.05% Tween20 [Sigma-Aldrich, St. Louis, USA] in Odyssey Blocking Buffer [LI-COR Biosciences, Lincoln, USA]) were added per well and incubated for 1 h. After removal of blocking solution, samples as described above and culture medium as blank were added with 100 µL per well and incubated at 4 °C overnight. The samples were removed, and arrays washed 3x for 5 min and 150 µL Wash Buffer I (Wash Buffer I concentrate 1:20 diluted in ultra-pure water) per well each. Each slide was submerged in Wash Buffer I 2x for 10 min each. The last two steps were repeated with Wash Buffer II (Wash Buffer II concentrate 1:20 diluted in ultra-pure water). 70 µL Biotin-conjugated anti-cytokines (stock reagent diluted with 300 µL blocking solution) were added per well and incubated for 2 h. Arrays were washed thrice with 150 µL Wash Buffer I and twice with 150 µL Wash Buffer II each for 5 min. IRDye^®^ 800CW Streptavidin antibody (1:3,000 in blocking solution, LI-COR Biosciences, Lincoln, USA) was added with 70 µL per Well and incubated for 45 min. Slides were finally washed four times with 150 µL Wash Buffer I, twice with 150 µL Wash Buffer II each for 5 min and with removed frame in provided centrifuge tube twice each with Wash Buffer I and II for 10 min each. All glass slides were scanned at 800 nm on Odyssey Clx with Image Studio software (LI-COR Biosciences, Lincoln, USA). The experiment was performed two times independently with biological duplicates per group.

### Generation of conditioned medium and assaying its influence on migration

Conditioned medium was generated like the supernatants for the secretome analysis. Briefly, 1.2 × 10^6^ senescent or non-senescent cells were seeded per T75 cell culture flask (Greiner AG, Kremsmünster, Austria). The following day, 20 mL fresh medium was added to each flask and 24 h later, the conditioned medium was transferred to a centrifuge tube (Greiner AG, Kremsmünster, Austria), centrifuged at 522×*g* for 5 min and the supernatant filtered with 0.2 µm syringe filters (VWR International, Radnor, USA) on SOFT-JECT^®^ syringes (Henke-Sass Wolf, Tuttlingen, Germany). The filtered conditioned medium was divided in aliquots and stored at − 80 °C.

To assess the influence of conditioned medium on the migration of healthy MSCs, the IncuCyte migration tool was used as described above. Therefore, only untreated low-passage MSCs were seeded in the insert plate and regular cell culture medium or conditioned medium was added to the reservoir plate. To reduce the potential problem of nutrient deficiency, also a mixture of 50% conditioned medium with 50% cell culture medium was used. The plates were recorded every 2 h for 188 h in total. The experiment was performed with 8 wells per condition and three times independently.

### Selective removal by senolytics

Non-senescent and senescent cells were seeded at 6,735 cells per well in a 96-well plate (Greiner AG, Kremsmünster, Austria) including medium controls each for background determination. The following day, the cells were exposed to increasing concentrations of senolytic drugs shown in Table S2 as well as solvent controls for 5 d or 24 h. Then cells were washed once with PBS and XTT staining solution (Sigma-Aldrich, St. Louis, USA) was prepared by mixing 5 mL XTT labeling reagent with 100 µL electron-coupling reagent per plate. Cells were incubated with 100 µL medium and 50 µL XTT staining solution and absorbance was determined at 450 nm with the reference set to 630 nm. Background absorbance was removed using wells only containing medium and viability was normalized to solvent controls. All experiments were performed with 4 biological replicates per group and three times (except 17-DMAG and dasatinib 24 h each two times) independently.

### Statistical analysis

Since the cells were isolated from different donors, differences between independent experiments somehow reflect variations derived from age, sex, and other parameters, which more closely resembles the biological nature than the use of immortalized cell lines or pools of donor materials. Overall, the results were still comparable between donors as previous studies showed (Schmidt et al. 2013).

Each experiment was repeated as indicated. The statistic software RStudio (version 1.2.1335 with R version 3.6.1) was used for statistical analysis and graphic presentation (RStudio Inc., Boston, USA). For viability tests, non-linear regression was performed and displayed as mean with 99% confidence interval. Means and smoothing function with 99% confidence intervals are displayed for representation of SA-β-gal and IncuCyte results. Otherwise, Tukey boxplots and single data points are shown. qPCR results are shown as means only for fold regulations > 2.0 or < -2.0 and *p* < 0.05. Senescent cells were compared to controls by the unpaired two-samples Wilcoxon test. *p* values < 0.05 were considered statistically significant.

## Availability of data and material

All data generated or analyzed during this study are included in this published article and its supplementary information.

## Supplementary Information

Below is the link to the electronic supplementary material.

Supplementary file1 (DOCX 736 KB)
