# Supplementary material for: Chronic senescent human mesenchymal stem cells as possible contributor to the wound healing disorder after exposure to the alkylating agent sulfur mustard
Source: Arch Toxicol. 2021 Jan 25;95(2):727–47. doi: 10.1007/s00204-020-02946-5 (PMC7870771; doi:10.1007/s00204-020-02946-5)
Supplement: Supplementary file 1 — Supplementary file1 (DOCX 736 KB) [file 204_2020_2946_MOESM1_ESM.docx]

Electronic Supplementary Material

**Chronic senescent human mesenchymal stem cells as possible contributor to the wound healing disorder after exposure to the alkylating agent sulfur mustard**

Simone Rothmiller, Niklas Jäger, Nicole Meier, Thimo Meyer, Adrian Neu, Dirk Steinritz, Horst Thiermann, Michael Scherer, Christoph Rummel, Aswin Mangerich, Alexander Bürkle, Annette Schmidt*

*Corresponding author

Bundeswehr Institute of Pharmacology and Toxicology, Neuherbergstraße 11, 80937 Munich, Germany; Universität der Bundeswehr München, Faculty of Human Sciences, Institute for Sports Sciences, Werner-Heisenberg-Weg 39, 85577 Neubiberg, Germany; annette.schmidt@unibw.de

**Suppl. Figure 1** Isolation, quality control, and viability after H_2_O_2_ exposure of MSCs. **a**-**h** Isolation process of human MSCs from the bone marrow of femoral heads. **a**-**c** Bone marrow was scraped from femoral heads using a sharp curette and MSCs were dissolved by agitation into culture medium. **d** The suspension was filtered through a 70 µm cell strainer and **e** density gradient centrifugation was performed using Ficoll^TM^ overlaid with the cell suspension. **f** MSCs are enriched in the interphase and **g** plated onto cell culture dishes after washing once with medium. After two days, a medium exchange was performed, **h** MSCs were further cultivated with medium exchange twice a week and passaged at about 60 % confluence. **i** Flow cytometric analysis of early-passage MSCs stained with five cell surface markers CD14-FITC, CD34-PE-Cy7, CD45-APC-Cy7, CD105-PerCP-Cy5.5, and CD106-APC shows 95 % mesenchymal stem cells defined as CD14^-^/CD34^-^/CD45^-^/CD105^+^/CD106^+^. **j** Adipogenic and osteogenic differentiation was performed for 14 or 21 days, respectively. After fixation, lipid droplets of adipocytes were stained with sudan III (orange) and nuclear counterstain with hematoxylin (blue). Calcified areas of osteocytes were stained with von Kossa (black) and nuclear counterstain with nuclear fast red (red). **k** MSCs were exposed to increasing H_2_O_2_ concentrations for 5 days, viability was assessed by XTT assay and normalized to corresponding solvent controls. LC_50_ of 322.7 ± 11.4 µM H_2_O_2_ was determined (n = 4 biological replicates per group from five independent experiments). Data are represented as linear regression including mean ± SD (red dots and black error bars), single data points (blue squares), and 99 % confidence intervals (red ribbon)

**Suppl. Figure 2** Senescence and apoptosis induction in MSCs by SM or H_2_O_2_ exposure. **a** Percentage of SA-β-gal positive MSCs 1, 7, 14, 21, and 28 days post exposure to SM, H_2_O_2_, or solvent control (n = three randomly selected fields per group from three independent experiments). **b** Apoptosis was determined by Annexin V green live staining and confluence by the area covered by cells using the IncuCyte microscope for 276 h in total every 2 h. After single dose exposure to SM concentrations from 10 to 500 µM, solvent (EtOH), and positive control (200 µM H_2_O_2_), confluence and the number of apoptotic cells were determined. Results are expressed as means and trend with 99 % confidence interval (n = three randomly selected images per group from three independent experiments). Data are represented as Tukey boxplots; * p < 0.05, ** p < 0.01, *** p < 0.001, **** p < 0.0001

**Suppl. Figure 3** Senescence induction by other agents. Representative images are shown. **a** Replicative senescence was determined by passaging control cells once a week until no replication occurred, which was observed with passage numbers of at least 13 (n = three independent experiments). **b** Long-term senescence was observed up to 24 weeks after exposure to 40 µM SM, whereas 10 µM SM and 200 µM H_2_O_2_ exposed cells were partially able to escape the senescence and start proliferating again (n = three independent experiments). **c** The same senescence inducing concentrations were used on commercially available adipose tissue derived MSCs and their medium. SM-induced senescence could be verified (n = two independent experiments). **d** Senescence could also be induced by a continuous SM exposure of 0.5 and 1 µM or 50 µM H_2_O_2_ every 2-3 days, while 0.1 µM SM was not sufficient (n = two independent experiments). **e** The single dose 40 µM SM exposure resulted in a similar increase in SA-β-gal positive cells over time, when compared with the different chemotherapeutic drugs cisplatin (20 µM), melphalan (50 µM) or bendamustine (50 µM) as well as after irradiation with 10 Gy (n = two independent experiments)

**Suppl. Figure 4** Analysis of senescent cells using flow cytometry. Green fluorogenic SA-β-gal substrate was used to stain senescent cells and representative microscopic images are shown in Fig. 2a. After cell detachment, 10,000 cells each were analyzed by flow cytometry. **a** The amount of SA-β-gal (FITC) positive cells was determined for senescent and non-senescent (control) MSCs three weeks post exposure and is shown representatively. **b** Three representative images of FITC negative control and FITC positive 40 µM SM exposed cells are shown. BF = brightfield (channel 1), FITC = (channel 2), SSC = Side scatter (channel 6). **c** Size determined by area mask of BF and granularity by bright detail intensity mask of SSC (n = three independent experiments). Data are represented as Violin plots and Tukey boxplots; * p < 0.05, ** p < 0.01

**Suppl. Figure 5** Secreted factors in cell culture supernatants. Cell culture supernatants were collected 24 h after cell seeding at different time points after SM, H_2_O_2_, or solvent (control) exposure. The concentration of 72 chemokines, cytokines, and growth factors was determined by Bio-Plex assays and normalized for the cell number. Data are represented as means of normalized concentrations and missing color indicates levels below the detection limit (n = biological duplicates per group from three independent experiments)

**Suppl. Figure 6** Differences in migration and scratch closure. **a** Using IncuCyte chemotaxis assay, different cells in FBS-reduced medium in inserts migrated towards the reservoir plate filled with standard medium. Images were recorded for 160 h in total every 2 h and for normalization, the confluence on the bottom was divided by the confluence on the top for each time point (n = up to 8 biological replicates per group from three independent experiments). **b** Increasing amounts of senescent cells were added to non-senescent controls and closure of a scratched area was observed (0 % = only non-senescent solvent controls, 10 % = 10 % senescent cells + 90 % controls, and so on). Scratch assay was performed using the wound maker and IncuCyte microscope. Cells were continuously observed for 234 h in total every 2 h, and the repopulated part of the initially wounded area was determined (n = up to 8 biological replicates per group from three independent experiments). **c** Influence of conditioned medium from non-senescent (control) or senescent cells on the migration of healthy MSCs. Conditioned medium was added into the reservoir plate (left) or diluted half and half with culture medium (right). Additionally, regular culture medium was added (orange, left). Images were recorded continuously every 2 h for 188 h in total and the confluence on the bottom was divided by the confluence on the top for each time point (n = up to 8 biological replicates per group from three independent experiments). Data are represented as means and trend with 99 % confidence interval

**Suppl. Figure 7** Additional senolytic drugs. The selectivity towards senescent cells of literature reported senolytic drugs was tested. Senescent and non-senescent (control) MSC were treated with increasing concentrations of **a** antimycin A, **b** etomoxir, **c** ABT-737, **d** quercetin, or **e** FOXO4-DRI for 5 days. Viability was assessed by XTT assay and normalized for each cell type to the corresponding solvent controls. Data are represented as linear regression including 99 % confidence intervals (n = 4 biological replicates per group from three independent experiments)

**Suppl. Table 1** qPCR results. Genes with fold regulation > 2.0 or < -2.0 and p values < 0.05 of all qPCR results

| **Gene** | **Fold regulation** | **p value** | **Cell type** |
| --- | --- | --- | --- |
| ACTB | -2.1 | 0.004 | 40 µM SM |
| BARD1 | -3.6 | 0.004 | 40 µM SM |
| BGLAP | 2.1 | 0.036 | 40 µM SM |
| BGLAP | 2.0 | 0.042 | 200 µM H_2_O_2_ |
| BRCA1 | -2.4 | 0.001 | 40 µM SM |
| BRIP1 | -4.8 | 0.002 | 40 µM SM |
| CASP1 | 2.2 | 0.045 | 40 µM SM |
| CCL1 | 2.1 | 0.001 | 40 µM SM |
| CCNA2 | -9.5 | 0.005 | 40 µM SM |
| CCNB1 | -11.4 | 0.006 | 40 µM SM |
| CDK2 | -2.6 | 0.027 | 40 µM SM |
| CDK7 | 2.2 | 0.016 | 40 µM SM |
| CDKN1A | 3.8 | 0.007 | 40 µM SM |
| CDKN1A | 3.5 | 0.005 | 40 µM SM |
| CDKN2A | 2.3 | 0.002 | 40 µM SM |
| CDKN2B | 5.1 | 0.002 | 40 µM SM |
| CDKN2C | -3.1 | 0.004 | 40 µM SM |
| CHEK1 | -2.5 | 0.005 | 40 µM SM |
| CHEK1 | -2.4 | 0.049 | 40 µM SM |
| COL3A1 | 2.2 | 0.016 | 40 µM SM |
| COL3A1 | 2.4 | 0.028 | 40 µM SM |
| CREG1 | 2.1 | 0.038 | 40 µM SM |
| CTSK | 2.0 | 0.047 | 40 µM SM |
| DDIT3 | 2.4 | 0.001 | 40 µM SM |
| EXO1 | 2.0 | 0.047 | 40 µM SM |
| EZR | -2.0 | 0.011 | 40 µM SM |
| FEN1 | -2.2 | 0.009 | 40 µM SM |
| GADD45A | 2.0 | 0.028 | 40 µM SM |
| GADD45G | 2.1 | 0.014 | 40 µM SM |
| H2AFX | -3.0 | 0.008 | 40 µM SM |
| IL26 | 2.3 | 0.041 | 40 µM SM |
| IL6ST | 2.6 | 0.029 | 200 µM H_2_O_2_ |
| ITGA2 | 3.1 | 0.040 | 40 µM SM |
| MDM2 | 2.7 | 0.023 | 40 µM SM |
| PMS1 | 3.1 | 0.040 | 40 µM SM |
| RAD51 | -3.7 | 0.013 | 40 µM SM |
| RBL1 | -2.3 | 0.020 | 40 µM SM |
| RND3 | 2.3 | 0.010 | 40 µM SM |
| SPARC | 2.2 | 0.005 | 40 µM SM |
| SPP1 | 3.5 | 0.001 | 10 µM SM |
| SPP1 | 3.5 | 0.013 | 40 µM SM |
| SPP1 | 6.6 | 0.001 | 200 µM H_2_O_2_ |
| TAGLN | -2.0 | 0.002 | 40 µM SM |
| TGFA | 2.0 | 0.006 | 40 µM SM |
| TIMP1 | 3.9 | 0.034 | 40 µM SM |
| TNFSF13 | 2.0 | 0.015 | 40 µM SM |
| XAB2 | -2.0 | 0.002 | 40 µM SM |
| XPA | 2.0 | 0.006 | 40 µM SM |
| XRCC2 | 3.9 | 0.034 | 40 µM SM |

**Suppl. Table 2** Senolytic drugs tested in XTT assay. The concentration of the stock solution, the solvent, and the final concentration range of each drug is shown

| **Drug** | **Stock solution** | **Solvent** | **Conc range** |
| --- | --- | --- | --- |
| 17-DMAG | 200 mM | DMSO | 6 pM – 90 µM |
| ABT-263 | 50 mM | DMSO | 0.1 – 250 µM |
| ABT-737 | 40 mM | DMSO | 0.05 – 400 µM |
| Antimycin A | 120 mM | EtOH | 3.9 – 2000 µM |
| Dasatinib | 160 mM | DMSO | 10 pM – 400 µM |
| Etomoxir | 100 mM | ultra-pure water | 3.7 – 1,900 µM |
| FOXO4-DRI | 4 mM | PBS | 0.3 – 130 µM |
| Quercetin | 50 mM | EtOH | 0.2 – 500 µM |
